# Select microbial metabolites promote tau aggregation in a murine tauopathy model

**DOI:** 10.1038/s41467-026-74775-6

**Published:** 2026-06-30

**Authors:** Sabeen A. Kazmi, Franciscus Chandra, Michael Wasney, Jenny Cheng, Gregory R. Lum, Malvika Iyer, Daria Di Blasi, Adriana N. Espinoza, Arlene Lopez-Romero, Xia Yang, Nandita Garud, Elaine Y. Hsiao

**Affiliations:** 1 Department of Integrative Biology and Physiology, University of California, Los Angeles, Los Angeles, CA USA; 2 Department of Ecology and Evolutionary Biology, University of California, Los Angeles, Los Angeles, CA USA; 3 UCLA Goodman-Luskin Microbiome Center, Vatche and Tamar Manoukian Division of Digestive Diseases, Department of Medicine, David Geffen School of Medicine, Los Angeles, CA USA

**Keywords:** Microbiome, Alzheimer's disease

## Abstract

The gut microbiome is emerging as a modifier of risk for neurodegenerative diseases, but underlying mechanisms remain poorly understood. Here, we show that the hTau.P301S mouse model for progressive tauopathy develops alterations in the composition and function of the gut microbiome that are not recapitulated in amyloid-based 5xFAD or 3xTg models for Alzheimer’s disease. Disrupting the gut microbiome via chronic antibiotic treatment exacerbates cognitive deficits and tau pathology in hTau.P301S mice, demonstrating a causal influence of the microbiome on tau-driven disease progression. This corresponds with widespread alterations in microbiome-dependent metabolites in the sera and brains of hTau.P301S mice, including subsets that correlate with the severity of tau pathology. By screening against tau biosensor cells, we identify select microbial metabolites—trimethylamine-N-oxide, 3-indoxyl sulfate, phenol sulfate, thymidine, and 2’deoxyuridine—that promote tau seeding and aggregation. Systemic administration of these metabolites worsens cognitive impairment and tau pathology in hTau.P301S mice. These findings establish a mechanistic link between the gut microbiome, serum and brain metabolites, as well as tau aggregation, suggesting that select microbial metabolites could potentially serve as therapeutic targets for tau-driven diseases.

## Abstract (web summary)

Here, using a mouse model of tauopathy, the authors reveal that disruption of the gut microbiome drives tau pathology and cognitive decline, and identify five microbial metabolites that directly promote tau aggregation, offering potential therapeutic targets.

## Introduction

Alzheimer’s disease (AD) arises from complex interactions between genetic, environmental, and lifestyle factors. Current treatments either provide some symptomatic relief without significantly altering disease progression or target only one of multiple pathological proteins, underscoring the urgent need for therapeutic strategies that more effectively address the complex and multifactorial etiopathogenesis of AD. Emerging evidence implicates the gut microbiome as a key modifier of AD progression due to its central role in regulating immune, metabolic, neurological, and aging-related processes^1^. Multiple human studies report alterations in the gut microbiome of AD patients relative to healthy controls^2,3^, which is similarly seen across various rodent models of AD^4^. The animal studies establish causality, wherein manipulating the gut microbiome modifies the severity of AD-related symptoms^5–8^. Consistent with this, a large human study found alterations in the gut microbiome of individuals with preclinical AD, prior to symptom onset^9^. These microbial signatures correlated with the severity of pathological biomarkers and enhanced computational predictions of preclinical AD status. This suggests that microbiome alterations occur early in the disease course and contribute to AD risk. Despite strong evidence from human and animal studies, the mechanisms linking microbial dysbiosis to core pathophysiological processes underlying AD risk remain unclear.

AD is characterized by both amyloid-β (Aβ) plaques and neurofibrillary tau tangles; however, microbiome studies to date have largely focused on amyloid pathology. Across multiple mouse models of amyloidosis, microbiome depletion via antibiotic treatment or germ-free rearing reduced Aβ plaque burden^5–8^ and glial reactivity^6,10^, suggesting that the microbiome promotes neuroinflammation. In contrast, few studies have examined the microbiome’s impact on tau pathology, which associates strongly with cognitive decline in AD and other tauopathies^11,12^. In a mouse model combining tauopathy and human apolipoprotein E (ApoE)—a major genetic risk factor for late-onset AD—microbiome deficiency decreased phosphorylated tau levels and glial activation, similarly implicating a microbiome-mediated neuroimmune mechanism^10^. Notably, this effect of the microbiome depended on the specific isoform of ApoE, suggesting that, in this mouse model, the microbiome promotes tau pathology through pathways involving lipid metabolism. Whether the disease-modifying effects of the microbiome extend broadly to other tauopathy models that lack genetically driven metabolic dysregulation remains unclear. While most studies implicate indirect neuroimmune modulation by the microbiome, increasing evidence indicates that microbial products, such as bacterial metabolites and amyloids, can directly altering the aggregation of misfolded proteins^13,14^. This raises the question of whether the microbiome may impact tau pathology by directly modifying tau aggregation.

Herein, we address these open questions by investigating the effects of the gut microbiome and gut microbial metabolites in the hTau.P301S (hTau) mouse model of progressive tauopathy and in tau biosensor cells for assessing tau seeding activity and aggregation. In hTau mice, we define alterations in the taxonomic composition, functional potential, and genomic adaptations of the gut microbiome over the course of disease progression. We assess causal roles for the gut microbiome in modifying the severity of central tau aggregation and cognitive deficits. We identify microbiome-dependent metabolites in the sera and brains of hTau mice, and screen for their influences on tau aggregation in vitro. Finally, we validate that a set of 5 microbiome-dependent metabolites exacerbates tau aggregation and cognitive decline when administered to hTau mice. This study uncovers fundamental insights into the molecular mechanisms by which the gut microbiome can regulate risk for tau-driven neurodegenerative diseases.

## Results

### hTau.P301S mice exhibit age- and genotype-dependent shifts in the gut microbiome

**Fig. 1:**
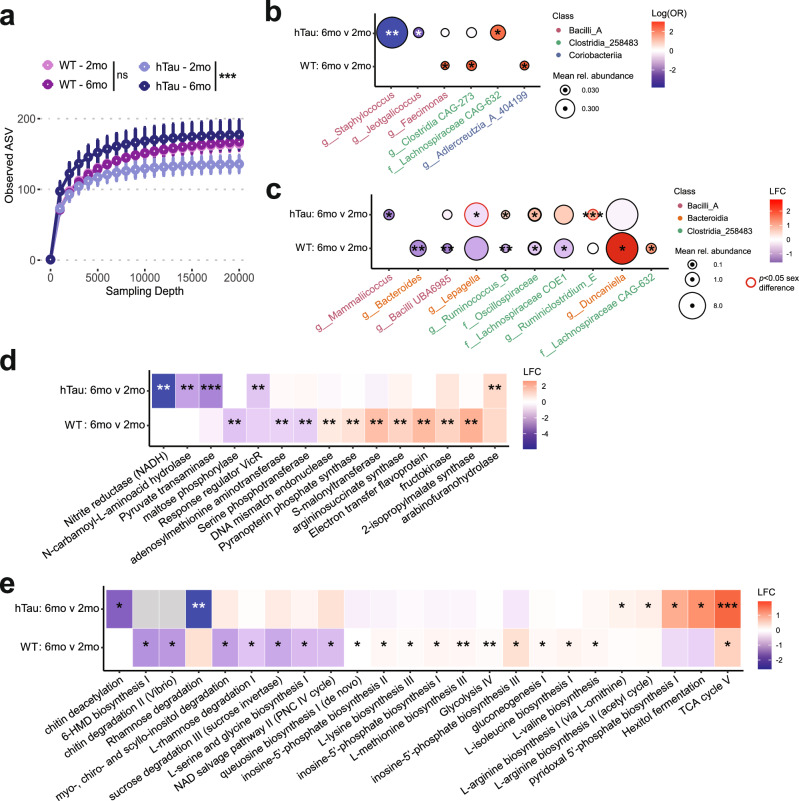
hTau.P301S mice exhibit aging- and genotype-dependent alterations in the gut microbiota. **a** Rarefaction curve of sequenced ASVs from hTau and WT SPF mouse fecal microbiota samples collected at 2 months (hTau *n* = 6; WT *n* = 8) and 6 months (hTau *n* = 7; WT *n* = 8; Kruskal-Wallis; *p* = 0.0005). **b** Differential presence/absence of microbes by logistic regression (*p* < 0.05) at 6 months (hTau *n* = 7; WT* n* = 8) compared to 2 months (hTau *n* = 6; WT *n* = 8) in hTau and WT mice. **c** Differential abundance of microbes by ANCOM-BC2 (*p* < 0.05) at 6 months (hTau *n* = 7; WT *n* = 8) compared to 2 months (hTau* n* = 6; WT *n* = 8) in hTau and WT mice. For (**b**) and (**c**), blank spaces denote the bacteria is not observed in both 2 months and 6 months and red outline indicates sex differences. **d** Gene families altered at 6 months (hTau *n* = 7; WT *n* = 8) compared to 2 months (hTau *n* = 6; WT *n* = 8) in hTau and WT mice (*p* < 0.05; Maaslin3 linear model with Benjamini-Hochberg procedure). **e** MetaCyc pathways altered at 6 months (hTau *n* = 7; WT *n* = 8) compared to 2 months (hTau *n* = 6; WT *n* = 8) in hTau and WT mice (*p* < 0.05; Maaslin3 linear model with Benjamini-Hochberg procedure). OR = odds ratio. LFC = log-fold change. Results in the rarefaction plot are expressed as mean ± SEM. *p < 0.05, **p < 0.01, ***p < 0.001, ****p < 0.0001, ns = not statistically significant.

Alterations in the gut microbiome have been reported in AD, but it is unclear if these changes occur through disease progression and aging, if they are driven by tau- vs. amyloid-based risk, and how they ultimately affect microbial function. To gain insight, we examined the gut microbiome over the course of disease in hTau mice, which overexpress tau, and compared them with signatures seen with aging in wildtype (WT) controls, in 5xFAD mice, which overexpress Aβ, and in 3xTg mice, which overexpress both tau and Aβ. By fecal 16S rRNA gene amplicon sequencing, hTau mice exhibited low numbers of observed amplicon sequence variants (ASV) at 2 months of age, prior to tau pathology, which significantly increased toward levels seen in WT controls by 6 months of age, when tau pathology and cognitive impairment are apparent (Fig. 1a and Supplementary Data 1)^15^. This increase in α-diversity may reflect a reduction in abundant bacterial taxa and enrichment of previously rare taxa to levels over thresholds for detection. There were no statistically significant differences between groups in Shannon diversity, Pielou’s evenness, and Simpson index (Supplementary Fig. 1a–c), suggesting that the alterations in α-diversity are primarily in community richness. In contrast, no differences in α-diversity were seen in the fecal microbiota of WT, 3xTg, or 5xFAD mice, indicating that the age-dependent shift in microbial richness observed in hTau mice is associated with its genotype under the specific housing and experimental conditions used (Supplementary Figs. 2a–d, 3a–d). There were also no global shifts in bacterial β-diversity, based on principal coordinates analyses of unweighted and weighted UniFrac distances, between 2 and 6 months of age for any genotype (Supplementary Figs. 1d, e, 2e, 3e). We next investigated differential presence versus absence based on the observed alterations in total richness. There were select genus-level shifts in hTau mice from 2 to 6 months, including significant decreases in *Staphylococcus* and *Jeotgalicoccus* and increases in *Lachnospiraceae* CAG-632, which were not seen in WT controls (Fig. 1b), 5xFAD mice (Supplementary Fig. 2f), or 3xTg mice (Supplementary Fig. 3f). Decreases in *Staphylococcus* were previously reported in the P301L tauopathy mouse model but were not observed in the 3xTg model (Supplementary Fig. 3f), suggesting that this microbial change may be associated with specific tauopathy models rather than representing a universal feature of tau-mediated neurodegeneration^16^. By differential abundance analysis, hTau mice exhibited decreases in *Lepagella* and *Mammalicoccus* and increases in *Ruminococcus_B, Oscillospiraceae, and Ruminiclostridium_E* from 2 to 6 months of age (Fig. 1c). hTau mice also exhibited a lack of aging-dependent increase in *Duncaniella* and decreases in *Bacteroides*, *Bacilli* UBA6985, and *Lachnospiraceae_*CAG-632, as seen in WT controls. Of these alterations, the aging-dependent decrease in *Lepagella* and increase in *Ruminiclostridium_E* were dependent on both the hTau condition and sex, suggesting only modest sex differences in hTau microbiome alterations. Overall, the limited number of differential taxa suggests that changes in microbial richness during disease progression in hTau mice may reflect variability in which microbial species are affected across individual animals.

To gain insight into potential functional changes, we performed deep metagenomic sequencing of WT and hTau fecal samples and found that microbial gene families related to nitrite reductase, N-carbamoyl-L-aminoacid hydrolase, and pyruvate transaminase were significantly decreased from 2 to 6 months of age in hTau mice, but not in WT controls (Fig. 1d and Supplementary Data 2, 3). Functional pathway analysis revealed significant increases in microbial pathways associated with hexitol fermentation, pyridoxal 5’-phosphate biosynthesis, TCA cycle V, and L-arginine biosynthesis in hTau mice, suggesting enhanced fermentative metabolism and adaptive energy production. In contrast, pathways involved in chitin deacetylation and rhamnose degradation were significantly decreased from 2 to 6 months, indicating reduced microbial capacity for processing specific polysaccharides (Fig. 1e and Supplementary Data 2, 3). These alterations were not seen in WT controls, suggesting that they reflect a disease-associated remodeling of gut microbial functional potential.

We next asked whether changes in genome-wide nucleotide diversity (π) in gut microbiota from 2 to 6 months are associated with the hTau or WT genotypes. We found no overall significant differences in the change in nucleotide diversity between the two genotypes (Supplementary Fig. 1f), implying that major differences in how diversity accrues in the gut microbiome are largely observed at the community level rather than the genetic level. Despite there not being a change in genome-wide nucleotide diversity, we did observe 159 single-nucleotide variants (SNVs) undergo extreme changes in allele frequency (from *f* ≤ 0.2 to *f* ≥ 0.8) between 2 and 6 months of age across both genotypes. Allele frequency changes of this magnitude over such short timescales are likely adaptive^17^. To understand whether certain changes in genetic variation over time were associated with the hTau or WT genotypes, we focused on genes that harbored SNVs that underwent extreme allele frequency changes between 2 and 6 months in multiple mice of the same genotype. This signal of parallelism could indicate potentially genotype-specific adaptations^18,19^. We found that 20 genes in a total of 10 species exhibited this parallelism between 2 and 6 months. 6 of these genes experienced parallel SNV changes in hTau mice (Supplementary Data 4) and 14 genes experienced parallel SNV changes in WT mice (Supplementary Data 5). These genes and the species harboring these genes were non-overlapping between the two genotypes. Genes that accrued SNV changes in hTau mice included DNA ligase and Peptidyl-prolyl cis-trans isomerase B, and genes that accrued SNV changes in WT mice included a carbohydrate diacid regulator, Undecaprenyl-phosphate 4-deoxy-4-formamido-L-arabinose transferase, HTH-type transcriptional repressor PurR, as well as others. Taken together, these results reveal genotype-dependent changes in the gut microbiome of hTau mice over the course of disease progression.

### Chronic disruption of the gut microbiome promotes cognitive deficits and tau pathology in hTau.P301S mice

**Fig. 2:**
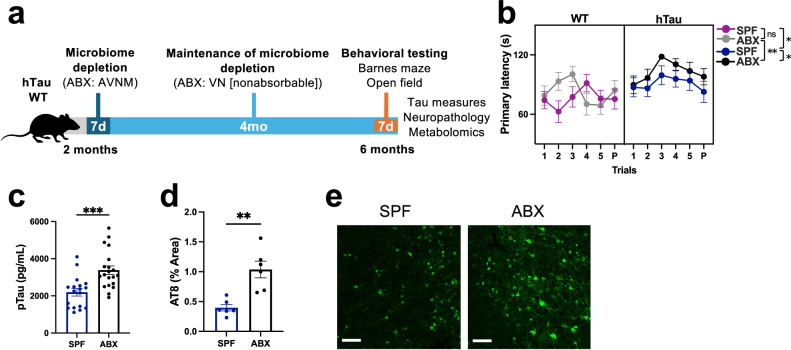
Depletion of the gut microbiota exacerbates cognitive behavior and tau pathology in hTau.P301S mice. **a** Experimental diagram for microbiome depletion. **b** Barnes maze performance for WT and hTau mice (WT ABX *n* = 19, SPF *n* = 20; hTau ABX *n* = 20, SPF *n* = 18). Primary latency to enter target hole (two-way ANOVA with Sidak correction; hTau ABX/SPF *p* = 0.0236; WT ABX/hTau ABX *p* = 0.0022; WT SPF/hTau SPF *p* = 0.0243). **c** Quantification of insoluble aggregated tau in 6-month-old brains of ABX (*n* = 20) and SPF (*n* = 18) hTau mice (two-tailed Welch’s *t* test; *p* = 0.0004). **d** Quantification of tau (AT8) pathology in the hindbrain of 6-month-old brains of ABX (*n* = 6) and SPF (*n* = 6) hTau mice (two-tailed Welch’s *t* test; *p* = 0.0043). **e** Representative images of AT8 staining of tau neuropathology in the pons of 6-month-old brains of ABX and SPF hTau mice (scale bars = 100 μm). Results are expressed as mean ± SEM. *p < 0.05, **p < 0.01, ***p < 0.001, ns = not statistically significant.

hTau mice develop progressive accumulation of phosphorylated tau and cognitive impairment from 4-6 months of age^20,21^. To gain insight into whether the microbial alterations seen in hTau mice influence the manifestation of AD-related outcomes, we chronically disrupted the gut microbiome by treating mice with oral antibiotics (AVNM: ampicillin, vancomycin, neomycin, and metronidazole) for 1 week at 2 months of age and further maintained them on non-absorbable antibiotics (VN: vancomycin and neomycin) in the drinking water until 6 months of age (Fig. 2a). This regimen caused an initial depletion toward germ-free status, which was maintained for at least 1 month, followed by a partial recovery of 16S rRNA gene burden, likely from resistant or tolerant bacteria, which was maintained through the end of the study (Supplementary Fig. 4a). At 6 months of age, antibiotic-treated (ABX) mice were assessed in the Barnes maze assay, a test for spatial learning and memory that is widely used to measure AD-related cognitive impairment. Mice were trained over 5 trials to identify which out of the 20 holes contains an escape box and then tested for their ability to recall the spatial location of the escape in a final probe trial 48 h post-training. Compared to WT controls, hTau mice that were conventionally colonized (specific pathogen-free, SPF) exhibited significantly increased primary latency to find the escape across all trials (Fig. 2b). This generally aligns with cognitive impairment in aged hTau mice, as reported previously^20,21^. Disrupting the gut microbiome via chronic ABX treatment exacerbated the behavioral deficits seen in hTau mice, where primary latency was further increased in ABX-treated hTau mice compared to vehicle-treated SPF hTau controls (Fig. 2b). This effect of microbiome disruption was not seen in WT mice, suggesting that ABX treatment worsens cognitive outcomes from the hTau genotype, rather than impairing cognitive performance in general. Improvement in escape latency across training days was modest, consistent with reports that shortened Barnes maze protocols often produce relatively shallow acquisition curves in younger WT mice while remaining sensitive to group differences^22^. In contrast, ABX treatment led to reduced locomotor activity in the open field in both hTau and WT mice (Supplementary Fig. 4b–e). Consistent with prior reports of behavioral alterations in this model, hTau mice also differed from WT controls in total distance traveled, velocity, and frequency of entries into the center zone under both SPF and ABX conditions (Supplementary Fig. 4b–e). Given that ABX treatment reduced locomotor activity similarly in both hTau and WT mice, but only impaired Barnes maze performance in hTau mice (and not WT), the differences in motor activity are unlikely to account for the genotype-specific cognitive deficits.

To quantify insoluble tau in whole-brain homogenates, tau was sequentially extracted into PBS-soluble, Triton X–soluble, and guanidine hydrochloride–extractable fractions, representing pools with increasing insolubility. ABX treatment significantly increased phosphorylated tau specifically in the guanidine-extractable fraction, which captures highly insoluble, detergent-resistant tau species not readily accessible by ELISA without strong denaturants, whereas no group differences were observed in PBS- or Triton-soluble fractions (Fig. 2c and Supplementary Fig. 4f). ABX-treated hTau mice also exhibited an increased burden of tau aggregates in the hindbrain, a site known to exhibit prominent tau pathology^15,23^, when compared to vehicle-treated SPF hTau controls (Fig. 2d, e). These findings indicate that ABX treatment is associated with both increased tau aggregation and impaired spatial learning in hTau mice. Although tau pathology in this model was most prominent in the pons and medulla, ABX-treated hTau mice exhibited increased latency to locate the escape hole in the Barnes maze, indicating impaired spatial memory performance. Together, these results show that microbiome depletion exacerbates both tau pathology and behavioral deficits in this model.

### The gut microbiome modulates serum and brain metabolites that are associated with tau burden

**Fig. 3:**
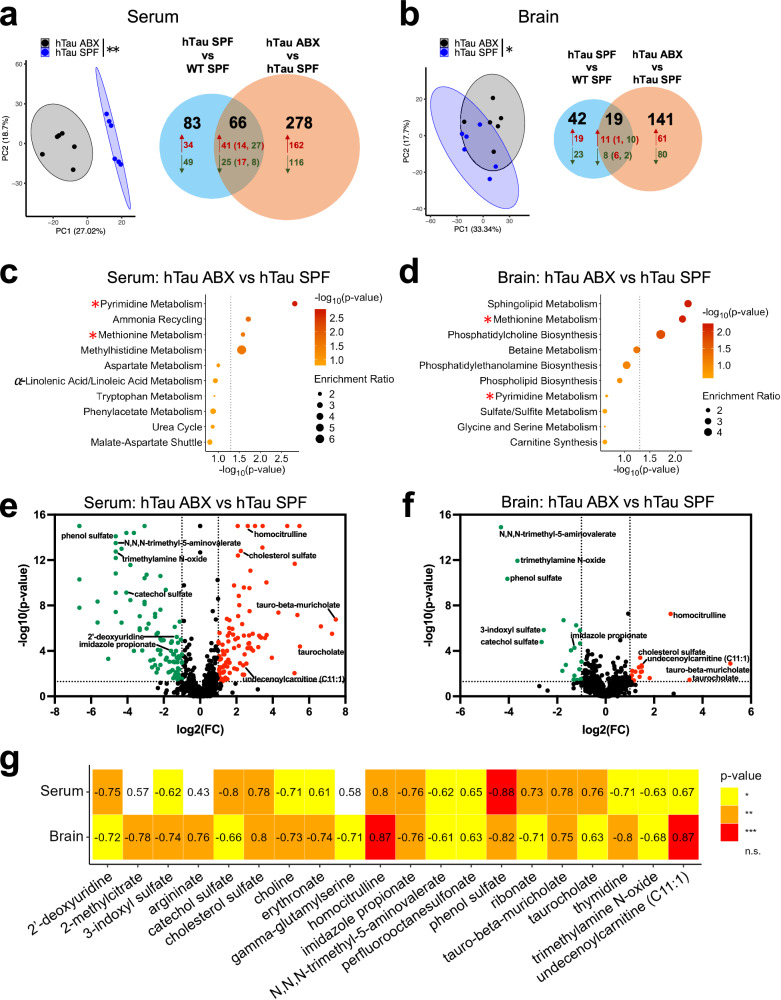
The gut microbiome regulates select metabolites in the serum and brain of 6-month-old hTau mice that correlate with tau aggregation. **a** Principal component analysis of serum untargeted metabolomics data from SPF hTau mice (*n* = 6) compared to hTau mice treated with ABX (*n* = 6; PERMANOVA; *p* = 0.002). Venn diagram denoting brain metabolites that were significantly altered comparing hTau SPF (*n *= 6) to WT SPF (*n* = 6) and hTau ABX (*n* = 6) to hTau SPF (*n* = 6). Number in the parentheses denote the difference in the directionality of significant metabolites in hTau/WT (blue circle) compared to their directionality in hTau ABX/SPF (orange circle) (*p* < 0.05, one-way ANOVA). **b** Principal component analysis of brain untargeted metabolomics from SPF hTau mice (*n* = 6) compared to hTau mice treated with ABX (*n* = 6; PERMANOVA; *p* = 0.041). Venn diagram denoting brain metabolites that were significantly altered comparing hTau SPF (*n* = 6) to WT SPF (*n* = 6) and hTau ABX (*n* = 6) to hTau SPF (*n* = 6). Number in the parentheses denote the difference in the directionality of significant metabolites in hTau/WT (blue circle) compared to their directionality in hTau ABX/SPF (orange circle) (*p* < 0.05, one-way ANOVA). **c** Top 10 enriched Small Molecule Pathway Database pathways (SMPDB) for serum metabolites in ABX-treated (*n* = 6) compared to SPF (*n* = 6) hTau mice (p < 0.05, one-way ANOVA). Asterisks for indicate pathways that are significant for both serum and brain. **d** Top 10 enriched Small Molecule Pathway Database (SMPDB) pathways for brain metabolites in ABX-treated (*n *= 6) compared to SPF (*n *= 6) hTau mice (*p* < 0.05, one-way ANOVA). Asterisks for indicate pathways that are significant for both serum and brain. **e** Volcano plot highlighting high fold-change serum metabolites from hTau mice significantly altered by ABX treatment. Metabolite candidates for in vitro testing highlighted in bold (*p* < 0.05, one-way ANOVA; ABX *n* = 6; SPF *n *= 6). **f** Volcano plot highlighting high fold-change brain metabolites from hTau mice significantly altered by ABX treatment. Metabolite candidates for in vitro testing highlighted in bold (p < 0.05, one-way ANOVA; ABX* n* = 6; SPF *n* = 6). **g** Correlation of brain and serum metabolites with levels of insoluble phosphorylated tau in the brain. Numbers denote Spearman’s rank correlation coefficients (ρ). Statistical significance was determined using a two-sided test (*p* < 0.05; ABX *n* = 6; SPF* n* = 6). *p < 0.05, **p < 0.01.

The gut microbiome regulates hundreds of known metabolites in the bloodstream and across several organ systems^24^. In light of the gut microbial alterations in hTau mice (Fig. 1) and the ability of microbiome disruption to promote tau pathology (Fig. 2), we hypothesized that the gut microbiome may promote disease progression via the regulation of microbial metabolites. To identify candidate metabolites that are microbially modulated and relevant to disease, we subjected serum and whole brain homogenates from ABX-treated hTau mice, SPF hTau mice, ABX-treated WT controls, and SPF WT controls to liquid chromatography tandem mass spectrometry (LC-MS/MS)-based metabolomic profiling. More than 6500 metabolites were detected in serum and brain (Supplementary Data 6, 7). Disruption of the gut microbiome with chronic ABX treatment led to widespread alterations in serum and brain metabolomic profiles, with statistically significant discrimination of ABX vs. SPF hTau conditions by principal components analysis (Fig. 3a, b). In particular, microbiome disruption by ABX yielded statistically significant alterations in 344 serum metabolites and 160 brain metabolites in hTau mice. Out of these differentially abundant metabolites, 66 (19%) of the serum metabolites and 19 (12%) of the brain metabolites were also differentially regulated in SPF hTau mice compared to WT controls (Fig. 3a, b). The relatively modest overlap suggests that chronic microbiome disruption may modify disease progression through mechanisms that are distinct from those caused by the genetic predisposition itself. Notably, compared to the effect of the hTau genotype relative to WT controls, chronic ABX treatment of hTau mice led to statistically significant alterations in ~ 2.3X more serum metabolites (344 [orange circle] vs. 149 [blue circle]) and ~ 2.6X more brain metabolites (160 vs. 61) (Fig. 3a, b and Supplementary Fig. 5), suggesting a larger modifying effect of the gut microbiome than for pathological tau expression. The differential serum metabolites related to pathways for the metabolism of pyrimidines, ammonia, methionine, and methylhistidine, whereas differential metabolites in the brain related to pathways for metabolism of sphingolipids, methionine, and phosphatidylcholine (Fig. 3c, d). The latter could relate to the role of sphingolipid metabolism on supporting cellular signaling and autophagy, methionine metabolism in regulating methylation status and oxidative stress, and phosphatidylcholine biosynthesis on enhancing neuroplasticity^25–27^. Furthermore, 42 metabolites were commonly differentially regulated in both the sera and brains of ABX-treated hTau mice compared to SPF hTau controls (Fig. 3e, f and Supplementary Data 8), suggesting that these particular microbial metabolites may cross the blood-brain barrier. Of these, 20 microbial metabolites in both sera and brain exhibited statistically significant correlations with brain levels of insoluble phosphorylated tau (Fig. 3g and Supplementary Fig. 7), suggesting that they regulate or respond to tau aggregation in the brain. These findings reveal striking effects of microbiome disruption on blood and brain metabolites, including subsets that are associated with the severity of tauopathy in hTau mice.

To assess whether these effects were genotype-specific, we analyzed antibiotic-treated WT mice relative to SPF WT controls. PCA revealed significant alterations in the serum but not the brain metabolome, and pathway analysis indicated partial overlap with several hTau ABX-altered pathways, including pyrimidine metabolism, the urea cycle, carnitine synthesis, and aspartate metabolism (Fig. 3a–d and Supplementary Fig. 6a–d). ABX treatment altered a subset of metabolites in both WT and hTau mice, including several that were similarly regulated across genotypes in both serum and brain (e.g., phenol sulfate, trimethylamine N-oxide [TMAO], and N,N,N-trimethyl-5-aminovalerate were decreased, whereas homocitrulline, cholesterol sulfate, taurocholate, and tauro-β-muricholate were increased in ABX mice), consistent with their dependence on the gut microbiome (Fig. 3e, f and Supplementary Fig. 6e, f). Conversely, other metabolites exhibited broader reductions across both serum and brain in hTau mice but were restricted to only the serum (thymidine and 2’-deoxyuridine) or the brain (3-indoxyl sulfate [3IS] and catechol sulfate) in WT animals. Given that WT mice do not develop measurable tau pathology, the functional relationship between these metabolites and tau aggregation can be assessed only in the hTau context. Together, these findings indicate that microbiome depletion induces shared peripheral metabolic changes, but their propagation to the brain may be dependent upon the presence of pathological tau.

### Select brain metabolites that are modulated by the gut microbiome promote tau aggregation and pathology

**Fig. 4:**
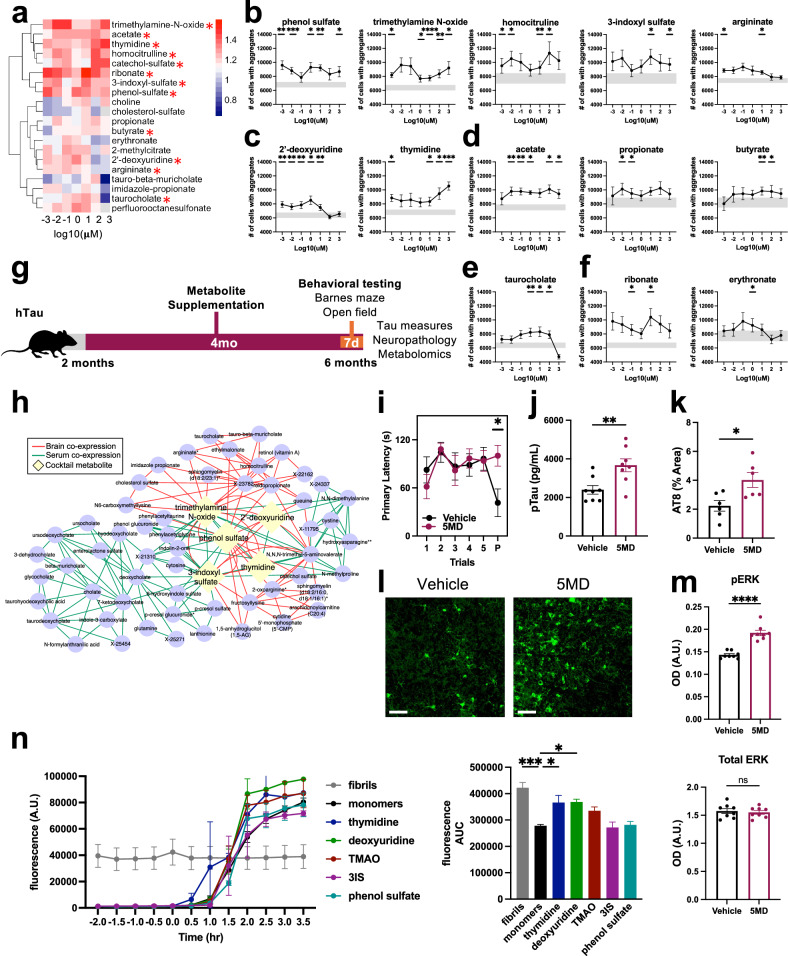
Select microbial metabolites promote tau aggregation and exacerbate tau pathology in hTau.P301S mice. **a** Effects of metabolites on tau aggregation in tau biosensor cells, expressed as percent cells that are aggregate-positive, normalized to brain homogenate + PBS control. Asterisks indicate statistically significant changes (one-way ANOVA with Dunnett’s test; *n* = 6 per metabolite per concentration). **b**–**f** In vitro dose–response curves for metabolites increasing aggregate-positive cells vs brain homogenate + PBS control. The shaded area shows baseline (brain homogenate + PBS control) ± SEM (one-way ANOVA with Dunnett’s test; exact *p-* values are provided in the Source Data; *n* = 6 per metabolite per concentration). **b** amino acid metabolites (**c**) nucleotide metabolites (**d**) short chain fatty acids (**e**) lipid metabolites (**f**) carbohydrate metabolites (**g**) Experimental diagram for metabolite supplementation (**h**) Metabolite co-expression module structures of brain module MEc1.16 and serum module MEc1.40. Network edge color denotes tissue (red for brain, green for serum). The five cocktail metabolites are highlighted by larger size and a diamond shape (Pearson correlation and Permutation; ABX* n* = 6; SPF *n* = 6) (**i**) Barnes maze performance for vehicle and mice treated with 5MD: 1.248 mM thymidine, 337.4 μM 2’-deoxyuridine, 1.930 mM TMAO, 2.715 mM 3IS, and 249.6 μM phenol sulfate. Primary latency to enter target hole (two-way ANOVA with Tukey’s test; *p *= 0.0176; vehicle *n *= 8, 5MD *n* = 8). **j** Quantification of insoluble aggregated tau in 6-month-old brains of vehicle and 5MD hTau mice (two-tailed Welch’s* t* test; *p *= 0.0079; vehicle *n* = 8, 5MD *n* = 8). **k** Quantification of tau AT8 pathology in the hindbrain (two-tailed Welch’s *t* test; *p* = 0.0193; vehicle *n* = 6, 5MD *n* = 6). **l** Representative images of AT8 staining of tau neuropathology in the hindbrain of 6-month-old brains of vehicle and 5MD hTau mice (scale bars = 100 μm). **m** Relative levels of pERK and total ERK in 6-month-old brains of vehicle and 5MD hTau mice (two-tailed Welch’s *t* test; *p* = <0.0001; vehicle *n* = 8, 5MD *n* = 8). **n** Effects of metabolites on heparin-induced aggregation of tau P301S monomers monitored by thioflavin T fluorescence (*n* = 3 per metabolite). Heparin was added at the 0 hr timepoint to induce fibrilization. Tau P301S fibrils were used as a positive control (one-way ANOVA with Dunnett’s test; monomers/fibrils *p* = 0.0002; monomers/thymidine *p* = 0.0140; monomers/deoxyuridine *p* = 0.0118). Results are expressed as mean ± SEM. *p < 0.05, **p < 0.01, ***p < 0.001, ****p < 0.0001, ns = not statistically significant.

Tau aggregation occurs through a self-amplifying seeding process that can be catalyzed or inhibited by small molecules^28–30^. To determine whether microbial metabolites found in the brain can modify tau aggregation, we screened top candidates (Fig. 3g and Supplementary Fig. 7) against tau biosensor cells, which express the aggregation-prone repeat domain of tau P301S fused with reporter proteins for fluorescence resonance energy transfer (FRET). At 48 hours after transfection with brain homogenates (BH) from hTau mice, tau aggregation was apparent by flow cytometry and microscopy for FRET signal, confirming adequate seeding from brain-derived tau fibrils (Supplementary Fig. 8a, b). Upon co-transfection with BH, 13 out of the 20 top candidate microbial metabolites significantly increased the count and proportion of FRET-positive cells in at least 1 out of 7 concentrations tested, spanning a physiological range (Fig. 4a, b–f). Of these, 9 microbial metabolites also induced significant increases in the integrated density of FRET signal (Supplementary Fig. 8c–g), suggesting that these metabolites may influence aggregate size, in addition to seeding activity. In particular, some metabolites (e.g., thymidine) elicited a clear concentration-dependent increase in aggregate-positive cells, whereas others (e.g., 2’ deoxyuridine) showed activity at lower concentrations that reduced with increasing levels (Fig. 4b–f), aligning with biphasic behavior previously reported^28^. Several microbial metabolites (e.g., TMAO, phenol sulfate, 3IS) showed bimodal response profiles, as previously seen with monoamines that promote fibril disaggregation^28^. Across all metabolites and concentrations included in the analysis, cell numbers remained above quality control thresholds ( > 20,000 events per sample) and no morphological evidence of cytotoxicity was observed, supporting the interpretation that FRET signal reflects tau aggregation rather than metabolite-induced cell death. While some metabolites exhibited non-linear or shallow dose–response curves, this behavior is consistent with previously reported biosensor assays and likely reflects factors such as cellular uptake, solubility, or aggregation kinetics, without affecting the overall interpretation of tau seeding and aggregation. These results reveal that select brain metabolites that are modulated by the gut microbiome and associated with elevated levels of insoluble tau aggregates in hTau mice have the capacity to directly promote tau seeding and aggregation in biosensor cells.

We next tested whether top microbial metabolites similarly exacerbate tau aggregation in vivo by administering them systemically to SPF hTau mice from 2 to 6 months of age (Fig. 4g). Metabolites were selected based on their (i) modulation by the gut microbiome, (ii) co-regulation in both the serum and brain, (iii) statistically significant association with brain levels of insoluble tau aggregates, (iv) ability to promote tau seeding activity and aggregation in vitro, and (v) their known presence in the human gut microbiome. These five metabolites were combined for in vivo treatment to test the cumulative effect of microbiome-derived metabolic perturbations, which is more physiologically relevant than testing individual compounds in isolation, as such combinations naturally occur in the gut. Metabolites were divided into two experimental groups: microbiome-dependent metabolites (5MD), which were decreased by ABX treatment and short-chain fatty acids (SCFAs), which are microbiome-dependent and previously implicated in AD^10,31^. In particular, network analysis of our full metabolomic dataset revealed notable co-expression of the five microbiome-dependent metabolites thymidine, 2’deoxyuridine, TMAO, 3IS, and phenol sulfate (5MD) in the serum and brain (Fig. 4h), with significant associations between their specific co-expression network as defined by their module eigengene and brain levels of insoluble phosphorylated tau (Supplementary Data 9). Mapping these metabolites to MetaCyc-annotated pathways identified candidate microbial producers in SPF hTau mice, including taxa involved in pyrimidine metabolism (Lactobacillus, Bifidobacterium, Lachnospiraceae), trimethylamine metabolism (Dorea, Parasutterella), and aromatic amino acid metabolism (Akkermansia, Acetatifactor), thereby linking community composition to metabolite production capacity (Supplementary Data 10). Together, these analyses connect microbiome composition to metabolite production capacity and provide a rationale to test their combined functional effects in vivo.

Metabolites were pooled and administered by intraperitoneal injection five times per week at physiological concentrations calculated based on our metabolomic data to reflect levels regulated by the microbiome and AD genotype. 5MD exacerbated cognitive deficits in hTau mice, as denoted by elevations in primary latency in the probe trial (Fig. 4i). This corresponded with significantly increased levels of insoluble phosphorylated tau in whole brain lysates of 5MD-treated hTau mice, as compared to matched vehicle-treated controls (Fig. 4j). Consistent with this, 5MD treatment led to a greater burden of tau aggregates in the hindbrain, as compared to vehicle-treated controls (Fig. 4k, l). In contrast, there were no significant effects of the SCFAs acetate, propionate, and butyrate on phosphorylated tau levels in hTau mice, as compared to their respective vehicle-treated controls (Supplementary Fig. 9g), suggesting specificity to the 5MD condition. Quantification of percent area covered by Iba1 and GFAP immunostaining revealed no differences between groups (Supplementary Fig. 9b, c), suggesting that 5MD exacerbates tau pathology through mechanisms that do not involve increases in gliosis and neuroimmune activation.

ERK phosphorylates tau at proline-directed sites, whereas GSK3β preferentially targets primed serine/threonine motifs. Together, these phosphorylation events are associated with reduced microtubule binding and promote tau aggregation. We hypothesized that 5MD may act intracellularly to promote tau hyperphosphorylation or directly enhance tau aggregation. To investigate potential influences of 5MD on intracellular signaling pathways that promote tau hyperphosphorylation, we measured levels of phosphorylated and total ERK and GSK3β. 5MD treatment selectively increased levels of phosphorylated ERK without altering total ERK, whereas both phosphorylated and total GSK3β were unchanged (Fig. 4m and Supplementary Fig. 9d), indicating that 5MD may promote tau aggregation by increasing ERK-dependent tau phosphorylation. To determine whether individual 5MD metabolites directly influence tau aggregation, we performed cell-free tau fibrillization assays. Prior in vitro work has demonstrated that metabolites such as TMAO can promote tau aggregation, but typically at highly supraphysiological concentrations (e.g., 200mM–1 M)^29^. In contrast, we tested metabolites at 200 µM, a concentration chosen to approximate the upper range of exposure achieved in our in vivo i.p. paradigm. Using a uniform concentration across metabolites enabled direct comparison of their aggregation-promoting effects under physiologically relevant conditions. Thymidine and 2’-deoxyuridine each significantly increased tau fibril formation, whereas TMAO, 3IS and phenol sulfate showed no statistically significant effect (Fig. 4n), suggesting that these two nucleoside metabolites in particular may drive the aggregation-promoting activity of 5MD. Collectively, these findings suggest that 5MD can increase tau pathology in vivo by promoting ERK-dependent tau hyperphosphorylation and directly exacerbating tau aggregation in a specific metabolite-dependent manner. Taken together, results from this study establish a causal role for the gut microbiome in modulating the severity of tauopathy and further identify key microbial metabolites in the blood and brain that catalyze tau aggregation in biosensor cells and exacerbate tau aggregation and cognitive impairment in hTau mice.

## Discussion

We find that genetic predisposition to tauopathy yields alterations in the gut microbiome over the course of disease progression and in metabolomic profiles in the systemic circulation and brain. Metagenomic profiling of the gut microbiome revealed distinct shifts in metabolic capacity in hTau mice compared to controls. In particular, we observed remodeling of microbial genes and pathways related to energy homeostasis, fermentative metabolism, nitric oxide production, and nitrogen cycling based on metagenomic data. These shifts suggest impairments to redox balance, nutrient cycling, and host–microbiome signaling, pathways that have been implicated in gut–brain interaction and Alzheimer’s disease progression^32,33^. The microbiome alterations corresponded with significant alterations in brain metabolites related to panthothenate and CoA biosynthesis and pyrimidine metabolism. Pantothenate, a CoA precursor, and CoA are critical for mitochondrial function and energy metabolism; disruptions in these pathways can result in mitochondrial dysfunction, a hallmark of AD^34^. In addition, alterations in pyrimidine nucleotide synthesis have been associated with cellular senescence, neuronal degeneration, and synaptic impairments^35^. A subset of metabolites, including N, N, N-trimethyl-5-aminovalerate and catechol sulfate, were dysregulated by the hTau genotype, microbiome-dependent, and co-modulated in both serum and brain, suggesting their ability to cross the blood–brain barrier (BBB). This points to the ability of pathological tau expression to alter the gut microbiome in ways that dysregulate metabolite levels in the brain and periphery.

We observed that further disruption of the gut microbiome via chronic antibiotic treatment led to worsened tau aggregation and cognitive performance in hTau mice, which corresponded with widespread alterations in serum and brain metabolomic profiles. Behavioral assessment relied primarily on the Barnes maze, a widely used assay of spatial learning and memory in Alzheimer’s disease mouse models, while complementary tests such as novel object recognition or Y-maze were not performed. The observed deficits are consistent with prior studies using the Barnes maze as a principal behavioral readout, and conclusions regarding cognitive impairment are framed with this limitation in mind^22,36–40^. Notably, the direction of change observed in this study, where antibiotic treatment exacerbates tau aggregation, contrasts a prior study of PS19-ApoE, 3xTg, 5xFAD, and APP/PS1 mouse models, in which acute antibiotic treatment generally reduced tau and/or Aβ pathology^6,7,10,41^. In addition to differences in the mouse line and antibiotic regimen, our chronic antibiotic treatment models persistent (rather than temporary) depletion of the gut microbiome, allowing us to evaluate its fundamental role over the course of disease. The ability to yield contrasting outcomes with different microbiome manipulations suggests that the gut microbiome acts as a disease modifier, rather than an etiopathological driver, that engages multiple parallel pathways of reducing or enhancing disease risk.

Furthermore, when comparing microbiome differences, in the PS19-ApoE model, antibiotic treatment decreased the relative abundance of SCFA-producing taxa, including members of the *Ruminococcaceae* and *Lachnospiraceae* families, accompanied by reduced SCFA levels and attenuation of neuroinflammation. By contrast, although microbiome profiling was not performed in antibiotic-treated hTau mice, differential abundance analysis of untreated animals revealed age-dependent increases in related Firmicutes lineages, including *Ruminococcus_B, Oscillospiraceae,* and *Ruminiclostridium_E*, alongside alterations in *Bacteroides* and *Lachnospiraceae*-associated taxa. Several of these taxa, including *Ruminococcaceae*-related lineages, show partial overlap with those implicated in PS19-ApoE mice, although directionality and context differ, likely reflecting differences in host genotype, age, and duration of microbiome perturbation. Consistent with this, SCFA administration did not modulate tau pathology in hTau mice, whereas other microbiota-derived metabolites identified in our study promoted tau aggregation. We highlight in particular thymidine, 2’deoxyuridine, TMAO, 3IS, and phenol sulfate, which are co-regulated in both blood and brain, catalyze tau seeding and aggregation in biosensor cells, and exacerbate tauopathy in hTau mice. The fact that these metabolites were downregulated with chronic antibiotic treatment, which also corresponded with worsened tau aggregation, suggests that they do not sufficiently explain the effects of chronic antibiotic treatment on exacerbating tauopathy in hTau mice. This may not be surprising, as the microbiome has myriad effects on host biology, including core metabolism, immune homeostasis, and neural function, which may all contribute to the adverse effects of microbiome depletion. With likely multiple mechanisms at play, our study isolates one potential pathway where particular microbial metabolites directly regulate cellular tau aggregation. Altogether, these studies indicate that while overlapping microbial lineages are observed across models, the functional consequences of microbiome perturbation are highly context-dependent. Importantly, specific microbial metabolites may provide a mechanistic link between the gut microbiome and tau aggregation, emphasizing the need to understand the various mechanisms at play in order to accurately predict whether a particular microbiome perturbation will influence disease risk- positively or negatively, if at all.

Previous studies using fecal or cecal microbiota transfer (FMT) have demonstrated that age- and AD-associated gut microbiomes can induce pathological changes in recipient animals^41–44^. In these FMT studies, recipient animals exhibited age- or AD-related alterations in cognitive behavior and amyloid or tau pathology, demonstrating the capacity of the gut microbiome to influence disease-related phenotypes. At the taxonomic level, they have reported changes such as decreased *Barnesiella* and *Prevotella*, increased *Rikenella*, altered *Enterobacteriaceae* and *Lactobacillaceae* abundance, and reductions in SCFA-producing bacteria such as *Lactobacillus* and *Akkermansia*. In our hTau model, we observed age- and genotype-dependent increases in *Ruminiclostridium_E, Ruminococcus_B*, and *Oscillospiraceae* and decreases in *Lepagella*, reflecting that while the specific taxa differ across models, microbiome alterations converge on the ability to influence disease-relevant phenotypes. While most work has focused on community-level effects, a few studies have begun to link FMT-induced changes to specific microbial metabolites, such as short-chain fatty acids, δ-valerobetaine, γ-aminobutyrate, taurine, and valine, suggesting that these molecules may mediate gut-to-brain signaling^13,45–47^. Of these, valine overlaps with our dataset, being elevated in the serum of antibiotic-treated compared to SPF hTau mice^46^. Butyrate was increased in FMT-treated transgenic mice, but was likely reduced in our ABX hTau mice, though they were not measured in our metabolomic panel^45^. Importantly, most FMT studies measured metabolites correlatively, with only δ-valerobetaine demonstrated to causally impair learning, memory, and neuronal activity in vivo^13^. Mechanistically, the prior metabolites are thought to act largely via neuroimmune regulation (SCFAs) or neuromodulation (GABA). Our work complements these findings by testing select microbial metabolites in mechanistic assays, showing that some metabolites can directly enhance tau fibrillation in vitro, increase pERK signaling in vivo, and influence tau pathology independently of overt gliosis. Together, these results provide a metabolite-focused framework for understanding how microbiome composition translates into modification of tau-related phenotypes, bridging community-level observations from FMT studies with molecular mechanisms of microbially derived metabolites.

Notably, our metabolites of interest—thymidine, 2’deoxyuridine, TMAO, 3IS, and phenol sulfate—were found to be primarily dysregulated by alterations in the gut microbiota rather than by the hTau genotype itself when compared to WT controls. This suggests that these metabolites, at homeostatic levels, may act in conjunction with genetically encoded increases in human mutant tau, to contribute to disease progression in hTau mice. Nucleosides from the diet, including thymidine and 2′-deoxyuridine, are actively metabolized by the gut microbiota and host. Imbalances in nucleosides, including thymidine and 2′-deoxyuridine, can contribute to oxidative stress and altered kinase/phosphatase activities^48^. TMAO is generated by microbial metabolism of dietary choline and L-carnitine to generate trimethylamine, which is absorbed into the host circulation and subsequently oxidized to TMAO by hepatic flavin-containing monooxygenases^49^. TMAO accelerates brain aging, synaptic dysfunction, and cognitive decline, and has been reported to promote amyloid plaques when administered to 5XFAD and APP/PS1 mice^50,51^. 3IS is generated by microbial metabolism of tryptophan to indole, which is subsequently hydroxylated and sulfated in the liver^52^. It is a uremic toxin that crosses the blood- brain barrier, promotes oxidative stress and inflammation, and sufficiently induces cognitive impairment and neurodegeneration in mice^53,54^. In cultured neurons, 3IS induces early apoptosis and reactive oxygen species^55^. Phenol sulfate is generated by bacterial metabolism of dietary tyrosine and similarly promotes oxidative stress as a uremic toxin^56^. Exactly how these microbial metabolites enhance tau aggregation is unclear, but there are multiple pathways of potential interest.

Small molecules can enhance tau aggregation through various mechanisms, including by inducing conformational changes that favor beta-sheet formation, stabilizing aggregation-prone conformations, cross-linking tau proteins, destabilizing tau-microtubule interactions, altering post-translational modifications, and inhibiting aggregate clearance. In a study of its biochemical interactions, TMAO altered the secondary structure of the C-terminal fragment of tau to promote its self-aggregation^29^. In addition, key kinases such as glycogen synthase kinase-3 (GSK3), mitogen-activated protein kinases (MAPKs), and cyclin-dependent kinase 5 (CDK5) have been implicated in tau phosphorylation dynamics^57,58^. Furthermore, several classes of microbial-modulated metabolites, including TMAO and 3IS, have been shown to alter MAPK signaling in cell lines and in vivo systems^59,60^. More broadly, prior studies have demonstrated that arginine residues on tau fibrils can engage in π-stacking interactions through its guanidinium group, promoting fibril stabilization and aggregation^61^. These observations align with our cell-based assays and in vivo validation, suggesting that certain microbiome-modulated metabolites, including thymidine and 2’-deoxyuridine, may act directly upon tau to promote aggregation and tauopathy in mice. In support of this, cell-free fibrillization assays showed that these two nucleosides significantly enhanced tau fibril formation at physiologically relevant concentrations, whereas the other three metabolites within the 5MD cocktail had no detectable effect. Building on these findings, we assessed whether microbiome-dependent metabolites influence tau phosphorylation in vivo. 5MD treatment exacerbated tau pathology in hTau mice, increasing insoluble phosphorylated tau and tau aggregate burden without significant microglial or astrocytic activation. Phosphorylated ERK was selectively increased in 5MD-treated mice, while total ERK and GSK3β were unchanged, suggesting that 5MD may promote tau phosphorylation through ERK-dependent mechanisms. Together, these results indicate that specific metabolites within 5MD can exacerbate tau pathology both through direct promotion of tau aggregation and selective activation of phosphorylation pathways, providing a mechanistic rationale for the observed in vivo effects. These findings provide a mechanistic framework that complements prior microbiome depletion and metabolite association studies, while laying the groundwork for future dissection of metabolite-specific pathways. Collectively, these observations suggest that microbial-modulated metabolites may promote tau pathology through multiple, potentially synergistic mechanisms—ranging from direct molecular interactions with tau to modulation of phosphorylation and proteostasis pathways.

Findings from this study provide proof-of-principle that genetic risk for tauopathy can yield progressive changes in the gut microbiome and that select microbially modulated metabolites in the serum and brain can promote tau aggregation in vitro and in vivo. They align with a growing number of human studies that report alterations in microbial metabolites in AD patients. For instance, excess TMAO has been linked to vascular dysfunction, oxidative stress, neuroinflammation, and tau phosphorylation in human AD patients^62,63^. 3IS has similarly been reported as elevated in patients with cognitive impairment and other neurological conditions, as compared to healthy controls^64,65^. A recent study found that microbial phenylacetyl-carnitine is positively associated with aging and cognitive impairment across multiple human AD studies^66^. Several metabolites of the kynurenine pathway, SCFAs, and bile acids have also been implicated in AD and positively correlated with dementia^67–71^. Overall, these findings reinforce the notion that microbial metabolites are associated with neurodegenerative processes and highlight the need to investigate basic mechanisms of microbial metabolite-tau interactions and further interrogate their potential relevance to human tauopathies. With increasing interest in microbiome-based interventions for neurodegenerative diseases, a deeper understanding of mechanistic links between gut microbes and hallmark neuropathological features is crucial for evaluating the potential to target specific microbes and their metabolites to slow tau-driven neurodegeneration.

## Methods

### Animals

5xFAD (B6.Cg-Tg(APPSwFlLon,PSEN1*M146L*L286V)6799Vas/Mmjax; Stock No. 034848, Jackson Laboratory), 3xTg (B6.Cg-Tg(APPSwe,tauP301L)1Lfa *Psen1*^*tm1Mpm*^/2 J; Stock No. 033930, Jackson Laboratory), and C57BL/6 J (Stock No. 000664, Jackson Laboratory) were purchased from Jackson Laboratory for this study. Homozygous hTau.P301S (B6-Tg(Thy1-MAPT*P301S)2541) mice backcrossed to a C57BL/6 J background were donated by the Goedert Lab (Medical Research Council Laboratory of Molecular Biology, Cambridge) and bred at the UCLA animal facility. The Institutional Animal Care and Use Committee at the University of California, Los Angeles, approved procedures used in this study. Animals were housed at an Animal Research Committee-accredited facility in compliance with the Guide for the Care and Use of Laboratory Animals. Mice were kept in standard environmental conditions and housed in autoclaved cages with a 12 h light and 12 h dark cycle with free access to standard chow (Lab Diet 5010) and water.

### Microbiome normalization

For each genotype, mice were randomly assigned to experimental groups at weaning. Each group contained equal numbers of age- and sex-matched male and female mice. Animals were housed two or three per cage per sex, and transgenic mice were not co-housed with non-transgenic or wild-type littermates during aging. Cage was treated as the biological unit in the experimental design and analyses, as coprophagic animals within a cage share a common microbiome. Accordingly, for microbiome profiling, reported sample sizes (n) reflect the number of cages per group rather than individual animals (20 mice). Microbiome normalization was performed via bedding exchange within each genotype, but not across genotypes. This approach was chosen to minimize the potential for microbiome convergence that could obscure genotype-specific effects, prevent the formation of hybrid microbial communities that do not naturally occur, and avoid unintended cross-genotype influences. During biweekly cage changes, soiled bedding was collected from each cage within a genotype and pooled. The pooled bedding was then redistributed across cages of the same genotype to normalize the microbiome across experimental groups. Bedding exchange was performed for 1 month prior to initiation of experiments. Subsequent microbiota analyses were performed to evaluate shifts with aging and disease progression within each genotype.

### Antibiotics treatment

A cocktail of broad-spectrum antibiotics consisting of vancomycin (5 mg/mL; Chem-Impex), neomycin (10 mg/mL; Sigma-Aldrich), and metronidazole (10 mg/mL; Sigma-Aldrich) was made using sterile water based on methods previously described to mimic GF status^1^. Mice were placed in sterile cages with autoclaved standard chow and subjected to antibiotic treatment to deplete the gut microbiota starting at 2 months of age via twice daily 200 μL oral gavage for one week. During this time, mice were given vancomycin (0.5 g/L; Chem-Impex), neomycin (1 g/L; Sigma-Aldrich), and ampicillin (1 g/L; Sigma-Aldrich) ad libitum in sterile water. Following the one-week treatment period, mice were transferred to a new sterile cage to avoid microbial contamination from fecal pellets and were maintained on the nonabsorbable antibiotics vancomycin (0.5 g/L; Chem-Impex) and neomycin (1 g/L; Sigma-Aldrich) in sterile water until euthanasia at 6 months of age. Cages, autoclaved chow, and water were changed weekly. For the vehicle-treated groups, sterile water was administered via oral gavage.

### Behavioral testing

All behavior testing was conducted at 6 months of age. Mice were monitored for signs of motor impairment prior to behavioral testing. Animals exhibiting overt hindlimb paralysis were excluded from behavioral analyses. None of the mice included in the behavior experiments displayed signs of hindlimb paralysis at the time of testing. Mice were removed from their home cages and placed in individual sterile holding cages. They were habituated to the testing room for 1 h prior to being subjected to behavior tasks. The testing room featured visual cues that were placed asymmetrically and varied in shapes and colors. Overhead lights and white noise were switched on during the testing periods. Mice were recorded using a ceiling-mounted Basler Gig3 camera and EthoVision XT software (v15; Noldus). The arenas were cleaned with 70% ethanol followed by Accel disinfectant before and after each session.

Open field testing was used to measure locomotion and anxiety-like behavior. The open field arena consisted of a 50 cm × 50 cm white PVC box with the center being a 15 cm square area. Mice were placed in the center of the arena and were allowed to freely explore for 10 min. Distance traveled and velocity were used to measure locomotion and exploration. The number of entries to the center and the duration of time spent in the center were used to measure anxiety-like behavior. Mice were returned to their home cage at the end of the task and allowed to rest for 1 h before being subjected to Barnes maze testing.

Barnes maze testing was used to assess cognitive deficits in learning and memory. The Barnes maze arena consisted of a circular white PVC slab (92 cm diameter, 95 cm height), with 20 evenly spaced holes (5 cm diameter) surrounding the perimeter. The arena was placed upon a rotating stool that contained the escape box. Testing procedures were adapted from Attar et al. 2013^2^. Briefly, Barnes maze testing took place over a 5-day period, starting with habituation (day 1), training trials (days 2-3), and a probe trial (day 5) that occurred 48 h after the last training trial. For the habituation phase, mice were removed from their holding cage and placed in a clear cylindrical container in the center of the arena for 30 s. They were then gently guided to the target hole that contained an escape box underneath over a 15 s period. Mice were given 3 min to independently enter the escape box. Those that failed to enter the escape box were guided in. Mice remained in the escape box for 1 min before they were returned to their home cage. The training phase consisted of 5 training trials over 2 days (3 trials on day 2 and 2 trials on day 3). For each training trial, the testing mouse was removed from the holding cage and placed in an opaque cylindrical container in the center of the arena for 15 s. The container was removed, and it was given 2 min to find the target hole containing the escape box. If it found the target hole and entered the escape box, it was allowed to stay in the box for 1 min. If it found the target hole but did not enter the escape box, it was placed in a clear cylindrical container at the target hole and given 3 min to independently enter the escape box. If the mouse did not independently enter the box, it was guided in and allowed to stay in the box for 1 min. If the mouse did not find the target hole, it was guided to the target hole using the clear cylindrical container, allowed 3 min to enter the escape box, and 1 min in the escape box after entering. The mouse was then returned to its holding cage. The process was repeated for the remaining training trials. Mice were returned to their home cage upon the completion of the last training trial of the day. For the probe trial, the escape box was removed from the target hole. The mouse again was placed in an opaque cylindrical container in the center of the arena for 15 s. It was given 2 min to freely explore the maze and locate the target hole. Upon completion of the probe trial, the mouse was returned to its home cage. Primary latency to the target hole was recorded for each trial.

### 16S rRNA Gene sequencing

Fresh fecal pellets were collected monthly from 2–6 months of age in a sterile 1.5 mL centrifuge tube and stored at − 80 ^o^C. Each n represents an independent cage containing 2-3 mice per cage to account for the effects of co-housing on normalizing the gut microbiota. DNA was extracted from fecal samples using the DNeasy 96 PowerSoil Pro kit (Qiagen). The sequencing library was generated following the Earth Microbiome Project’s 16S Illumina Amplicon Protocol using forward and individually barcoded reverse primers that were created by Caporaso et al. 2011^3^. PCR amplification of the V4 region of the 16S rRNA gene was done in triplicate using 30 ng of the bacterial genomic DNA. The triplicates of the PCR products were pooled and ran on a 1% agarose gel to confirm the presence of the amplicon (approximately 390 bp). Upon confirmation, PCR products were purified using the QIAquick 96 PCR Purification kit (Qiagen). Samples were sequenced on the Illumina MiSeq platform using a 2 × 250 bp reagent kit for paired-end sequencing by Laragen, Inc. DADA2 was used for quality filtering, quality control, and amplicon sequence variants (ASVs) identification. ASVs were assigned using a naïve bayes classifier trained on the full-length 16S greengenes2 database (release 09.2024). DADA2 and taxonomy assignment was done within QIIME2 2025.4 environment^4^. Alpha and beta diversity analysis were done using the vegan library (v2.7.1). For alpha diversity measurements, all samples were first rarefied to 20000 reads. Differential abundance of genus-level feature counts was conducted using ANCOM-BC2 (v2.8.1) with fixed_variables = mouse sex + sampling timepoints and prevalence_cut = 0.2. Features that were labeled as structural zeroes by ANCOM-BC2 were selected and analyzed for differential presence/absence using a logistic regression model in the logistf library (v1.26.1). Plots were generated using ggplot2 (v3.5.2) and ggpubr (v0.6.1). Alpha diversity, beta diversity, differential abundance analysis, and plotting were conducted in R (v4.4.1).

### 16S rRNA Gene load quantification

Depletion of the microbiome was validated by quantitative PCR (qPCR) measurement of 16S rRNA gene loads in fecal samples of antibiotic treated mice. qPCR with reverse transcription (qRT–PCR) was performed on the QuantStudio 5 thermocycler (ThermoFisher Scientific). SFP and GF fecal DNA were used as controls. qPCR was performed using validated primer sets from the BactQuant assay and SYBR green master mix with Rox passive reference dye.

### Metagenomic profiling and analysis

Fecal DNA was fragmented and barcoded using the Illumina DNA Prep kit (San Diego, CA) following the manufacturer’s instructions. The barcoded shotgun libraries were sequenced on an Illumina NovaSeq X Plus system using 25B flowcells and a 2 × 150 base pair (bp) sequencing configuration as previously described in Sato et al. 2019^6^. The raw sequence reads were processed using KneadData for quality filtering and removal of mouse-derived reads. The filtered reads were then analyzed using the following bioinformatics tools for taxonomic composition and functional assessments^7^. First, MetaPhlAn4 was used to identify the bacterial species present in the samples and determine the compositional profiles of the microbial communities^8^. Taxonomic analysis using metagenomic data rendered fewer known species maps, compared to known maps from 16S rRNA gene sequencing data (to the Greengenes2 database). We therefore focused metagenomic data analysis on functional potential, and performed taxonomic analysis using 16S rRNA gene sequencing data. Next, HUMAnN3 was employed to annotate bacterial genes and determine their functional roles and metabolic pathways. The bacterial gene abundances were aggregated into metabolic pathways based on the MetaCyc pathway classifications^9^. Differential abundance of log-transformed KO genes or pathway counts was conducted using Maaslin3 (v3.0.1) with fixed_formula = mouse sex + sampling timepoints and min_prevalence = 0.2. Plots were generated using ggplot2 (v3.5.2) and ggpubr (v0.6.1). Differential abundance analysis and plotting were conducted in R (v4.4.1).

To ascertain single-nucleotide variants from the metagenomic data, metagenome assembled genomes (MAGs) were co-assembled using a previously established protocol^72^. Co-assembly was performed on a per-mouse basis using all samples belonging to the same mouse (i.e., 2-, 4-, and 6-months samples). A reference database consisting of dereplicated MAGs was constructed with MIDAS (v3.0.1)^73,74^. MIDAS was subsequently used to ascertain species abundance, single-nucleotide variants (SNVs), and gene content. π was calculated using a formula previously developed in Schloissnig et al., 2013^75^.

1 $$\pi (S,G)=\frac{1}{|G|}{\sum }_{i\,=\,1}^{{|G|}}{\sum }_{{B}_{1}\in \{{ATGC}\}}{\sum }_{{B}_{2}\in \{{ATGC}\}{{\rm{\backslash }}}{B}_{1}}\frac{{x}_{i,{B}_{1}}}{{D}_{i}}\frac{{x}_{i,{B}_{2}}}{{D}_{i}-1}$$

where S is the sample; G is the genome of the focal species, with ${|G|}$ being its size; i is the locus in the genome; ${B}_{1}$ and ${B}_{2}$ are the reference and alternative alleles, respectively; ${x}_{i,{B}_{j}}$ is the number of reads with allele ${B}_{j}$ at locus i; and ${D}_{i}$ is the total read depth at locus i. A Wilcoxon rank sum test was used to assess whether changes in π from 2 to 6 months within hosts was significantly different between the hTau and WT genotypes.

SNVs undergoing extreme allele frequency changes were defined from *f* ≤ 0.2 to *f* ≥ 0.8 between the 2- and 6-months samples in strains that were quasi-phaseable, as outlined in Garud et al., 2019^17^. When identifying genomic variants undergoing extreme frequency changes, only genomic loci with a read depth of ≥ 20 reads were considered in both the 2- and 6-months samples. To avoid confounding SNV changes due to de novo mutation vs strain fluctuations or large horizontal gene transfer events, only time point pairs with 20 or fewer SNV changes were analyzed. Genes experiencing parallelism were defined as harboring one or more SNVs undergoing an extreme allele frequency change in multiple mice of the same genotype. Gene descriptions were obtained from the MIDAS database that was constructed above, and KEGG ids were obtained by running the corresponding amino acid sequence for the gene in the representative genome for that species through BlastKOALA^76^. Python (v3.8.18) was used for all analyses, including the generation of tables. Graphs were generated in R (v4.4.1) using ggplot2 (v3.5.2) and ggpubr (v0.6.1).

### Tissue collection

Mice were anesthetized by inhalation of isoflurane, after which the heart was accessed through the opening of the abdominal cavity. Blood was collected from the right ventricle using a 26-gauge needle and stored on ice in serum-separating tubes (BD Vacutainer). Serum was separated by centrifugation at 1500 rpm at 4 ^o^C for 10 m. Serum was transferred into cryovials and flash frozen. For tissue collection, mice were transcardially perfused with ice-cold 1 × calcium- and magnesium-free Dulbecco phosphate-buffered saline (DPBS; Thermo Fisher). Brains were collected and transected into two hemispheres. The left hemisphere for all mice was flash frozen for tau quantification. For half the mice in each experimental group, the right hemisphere was flash frozen for metabolomic profiling. For the other half of the mice, the right hemisphere was post-fixed in 4% paraformaldehyde (PFA) for 24 h at 4 ^o^C. All frozen tissues were stored at − 80 ^o^C.

### Tau quantification

A 10% (wt/vol) brain homogenate was prepared by homogenizing frozen left hemispheres in ice-cold 1 × DPBS using the 4-Place Mini Bead Mill Homogenizer (VWR). Brain homogenates were aliquoted and stored at − 80 ^o^C. Total protein concentration was determined for each brain homogenate using the Rapid Gold BCA Protein Assay kit (Pierce). Protein concentration was standardized to 5 mg/mL with cold 1 × DPBS. Samples were centrifuged at 20,000 × *g* for 20 min at 4 ^o^C. The supernatant was collected as the soluble fraction, and the pellet was resuspended in cold 10% Triton X-100. Samples were incubated in ice with vortexing every 5 min for 20 min and then centrifuged at 20,000 × *g* for 20 min at 4 ^o^C. The supernatant was collected as the membrane bound fraction, and the pellet was resuspended in 5 M guanidine hydrochloride. Samples were incubated for 2 h at room temperature with shaking at 1000 rpm. The molarity was adjusted to 1 M guanidine using ice-cold 1 × DPBS. Samples were then centrifuged at 20,000 × *g* for 20 min at 4 ^o^C. The supernatant was collected as the insoluble fraction, and the pellet was discarded. Quantification of insoluble phosphorylated tau in the brain were quantified by performing enzyme-linked immunosorbent assay (ELISA) was performed using the Human Tau (Phospho) [pS199] (Invitogen) according to the manufacturer’s instructions.

### Immunofluorescence staining

Post-fixed brains were cryopreserved in 30% sucrose in 1 × DPBS for 24 h at 4 ^o^C. Cryopreserved brains were frozen on dry ice and cut into 20 μm thick coronal serial sections using a Leica CM1950 cryostat. Sections were collected in 1 × DPBS. Every sixth brain section from rostral to caudal (bregma coordinates −3 mm to − 7 mm) were used for immunofluorescence staining. Sections were washed and permeabilized in 0.5% Triton/0.05% tween-20 in 1 × DPBS (PBS-TT) three times for 15 min each. Afterward, sections were blocked with 5% normal goat serum (NGS) in PBS-TT for 2 h at room temperature. Sections were incubated at 4 ^o^C with primary antibodies diluted in blocking solution (5% NGS + PBS-TT). Brains were stained with Phospho-Tau (Ser202, Thr205) AT8 (biotin; 1:200; ThermoFisher), anti-Iba-1 (rabbit; 1:500; Wako) and GFAP (chicken; 1:500; ThermoFisher). 24 h later, sections were washed with PBS-TT four times for 15 min each prior to incubation with secondary antibodies in blocking solutions at room temperature in the dark for 2 h. Sections were stained with Alexa Fluor 488 (Streptavidin; 1:1000; ThermoFisher), Alexa Fluor 750 (goat anti-rabbit; 1:1000; ThermoFisher), and Alexa Fluor 555 (goat anti-chicken; 1:1000; ThermoFisher). Sections were washed with PBS three times for 5 min each prior to being mounted on slides using the ProLong Diamond Antifade Mountant with DAPI (Invitrogen). Whole-mount slide scanning images of brain sections were obtained using PhenoImager HT (Akoya Biosciences). At least three technical replicates per slide were used for analysis done with QuPath (v5.1; Bankhead et al. 2017)^77^. All quantification analysis were performed by a researcher blinded to the experimental groups. A pixel intensity threshold was established for each antibody and consistently applied across all slides within each experimental study. The percent area of antibody-positive pixels was quantified in the hippocampus, entorhinal cortex and hindbrain. For the hindbrain analysis, the pons and medulla were combined and analyzed as a single region of interest.

### Brain and serum metabolomic profiling

A total of six ABX and six SPF hTau mice, together with age-matched WT controls, were included. Brain and serum were collected from the same animals. Each group comprised three biological replicates per sex (*n* = 6 per group), and all samples were from independent animals with no technical replicates excluded. All samples were run on a Waters ACQUITY ultra-performance liquid chromatography (UPLC) and a Thermo Scientific Q-Exactive MS interfaced with a heated electrospray ionization (HESI-II) source and Orbitrap mass analyzer by Metabolon, Inc. Samples were prepared using the automated MicroLab STAR system (Hamilton Company). To form the metabolite extract, samples were extracted with methanol to precipitate protein and dissociate small molecules. The extract was split into four fractions, concentrated using a TurboVap system (Zymark) and stored in liquid nitrogen for analysis. Each fraction was analyzed on a separate arm of the platform and ran with a series of standards at fixed concentrations to ensure injection and chromatographic consistency. The first three fractions were eluted from a C18 column, while the fourth was eluted from a HILIC column. The first fraction was eluted using acidic solvents suitable for hydrophilic compounds and analyzed using UPLC-MS/MS with positive ion mode electrospray ionization (ESI). The second fraction was eluted using acidic solvents suitable for hydrophobic compounds and analyzed UPLC-MS/MS with positive ion mode electrospray ionization (ESI). The third fraction was eluted using basic solvents and analyzed using RP/UPLC-MS/MS with negative ion mode ESI. Lastly, the fourth fraction was eluted using polar solvents and analyzed UPLC-MS/MS with negative ion mode ESI. Chemical entities were identified by comparison to metabolomic library entries of purified standards. Metabolite peaks were quantified as area-under-the-curve detector ion counts. For studies spanning multiple run days, data were block-corrected to account for inter-day instrument variability by scaling daily medians to 1.00 and proportionally adjusting individual measurements. Standard statistical analyses were performed in ArrayStudio using log-transformed data. Differentially abundant metabolites between groups were identified using one-way ANOVA, and *p*-values were calculated to assess statistical significance. The false discovery rate (FDR) was estimated using the q-value method previously published^78^. Briefly, *p*-values derived from the one-way ANOVA and associated contrasts were ordered from smallest to largest, and tests with *p* < 0.05 were considered statistically significant. For this subset of results, *q*-values were computed, providing an estimate of the expected proportion of false positives among all tests declared significant at this *p*-value threshold. Principal components analysis (PCA) was used to visualize the distribution of variance across samples. Metabolite set enrichment analysis (MSEA) using the Metaboanalyst 6.0 platform was performed on mouse brain and mouse serum metabolites statistically significantly altered in ABX-treated mice compared to SPF within each AD mouse line or wildtype controls (non-adjusted *p*-value < 0.05). Metabolite sets were analyzed for chemical subclass enrichment and metabolite pathway enrichment, using the Small Molecule Pathway Database (SMPDB). PCA plots were generated in R using ggplot2 (v3.5.1) and vegan (v2.6.6.1).

### Metabolite coexpression module construction and association testing

Multiscale Embedded Gene Co-expression Network Analysis (MEGENA) was used to construct tissue-specific metabolite coexpression networks from mouse brain and serum metabolomics^79^. Metabolite coexpression modules were identified using a shortest path distance (SPD)-based distance measure. Nested k-medoids clustering that iteratively defines k-best clusters to minimize intra-cluster SPD was used until no more compact child clusters could be formed. Module eigengenes were calculated using the Weighted Gene Co-expression Network Analysis (WGCNA) moduleEigengenes^80^. *T* test or ANOVA was used to determine significant associations between coexpression metabolite modules and traits.

### Tau aggregation assay

Aged hTau brains were pooled, and a 10% (wt/vol) pooled brain homogenate (BH) was prepared by homogenizing frozen brains in ice-cold DPBS using the 4-Place Mini Bead Mill Homogenizer (VWR). Total protein concentration was measured using the Rapid Gold BCA Protein Assay Kit (Pierce), and protein concentration was standardized to 5 mg/mL using DPBS. Brain homogenates were aliquoted and stored at − 80 ^o^C for use in cell assay. Tau RD P301S FRET Biosensor cells (ATCC, cat# CRL-3275) were plated at a density of 35,000 cells in 120 μL of media per well in a 96-well plate. Cells were incubated at 37 ^o^C in a humidified atmosphere of 5% (vol/vol) CO_2_ for 24 h to allow the cells to adhere to the plate. Samples were prepared by combining metabolites (final concentration in the wells: 1 mM, 100 μM, 10 μM, 1 μM, 100 nM, 10 nM, and 1 nM) and BH (final concentration in the well 5 μg/mL) in DPBS. Liposome-mediated transduction complexes were made by combining 15 μL of sample with 14.17 μL of Opti-MEM (Gibco) and 0.83 μL of Lipofectamine 2000 (Invitrogen). Transduction complexes were incubated at room temperature for 25 min prior to adding to cells. Plates were covered with a porous adhesive film (VWR), and cells were incubated with the transduction complexes for 48 h at 37 °C in a humidified atmosphere of 5% (vol/vol) CO_2_ prior to flow cytometry. For technical replicates, samples were plated in triplicate per plate. Six plates using different passaged cells made up the biological replicates.

Cells were visually inspected by microscopy prior to flow cytometry to confirm adherence and normal morphology. Washing steps removed non-adherent cells, effectively excluding dead or dying cells from analysis. During assay optimization, certain compounds (including methylene blue, hemin chloride, and quinacrine dihydrochloride) produced substantial reductions in cell number at high micromolar to millimolar concentrations, evident by low event counts ( < 10,000 events) and abnormal morphology. Any sample that exhibited cell counts below 20,000 events or morphological evidence of compromised cell health was excluded from analysis. For all metabolites reported in this study, cell numbers exceeded 20,000 events per sample and morphology remained normal, supporting the interpretation that FRET signals reflect tau aggregation rather than cytotoxicity. Cells were harvested with 0.05% trypsin and resuspended in DPBS. The Attune NxT Flow Cytometer (ThermoFIsher) with the CytKick Autosampler (ThermoFisher) was used to perform FRET flow cytometry. To measure CPF and FRET, cells were excited with the 405 nm laser, and fluorescence was captured with the 440/50 nm and 512/50 nm filters. To measure YFP cells were excited with a 488 nm laser, and fluorescence was captured with a 525/50 nm filter. To quantify the FRET signal, a bivariate plot of FRET vs. CFP was created. A triangular gate was introduced to quantify FRET-positive cells. The FRET gate was adjusted using cells that received transduction complexes made of PBS only to exclude FRET-negative cells. 20,000 cells per well were analyzed for each experiment. The raw counts, percentage of FRET-positive cells and geometric mean fluorescence intensity were recorded. Integrated FRET density was calculated by multiplying the percentage of FRET-positive cells by the geometric mean fluorescence intensity. Data analysis was performed using FlowJo (v10).

### Metabolite supplementation

hTau mice were randomly assigned to two experimental groups and two control groups. Each group contained equal numbers of age- and sex-matched cohorts of males and females. The microbiome was normalized in the same manner as stated above prior to the start of the experiment. To test the effects of microbially modulated metabolites on the pathogenesis of AD, metabolites were split into two experimental groups: microbiome-dependent (5MD: metabolites lowered by ABX treatment), and short-chain fatty acids (SCFAs: produced by bacterial fermentation as a specific subclass of MD metabolites). Each experimental group had its respective vehicle group that was administered to provide matching sodium and/or potassium concentrations. Serum metabolomic data (AD vs WT and AD ABX vs AD SPF) and physiological concentrations reported in the literature were used to calculate metabolite levels that needed to be achieved daily in the serum. Metabolite concentrations were calculated based on its physiological levels in a WT mouse (or human in cases where mouse metabolite levels were undetermined), the difference between AD vs WT, and the relative elevation or reduction observed in the serum of AD ABX mice compared to AD SPF controls (Supplementary Data 6, 7). The metabolite stock solution was formulated by taking account the total blood volume of average mouse weighing 25 mg (approximately 58.5 mL/kg)^13^. Stocks for 5MD metabolites consisted of 1.248 mM thymidine, 337.4 μM 2’-deoxyuridine, 1.930 mM TMAO, 2.715 mM 3IS, and 249.6 μM phenol sulfate. Stocks for SCFAs consisted of 35.04 mM acetate, 3.154 mM propionate and 3.154 mM butyrate. All stocks were made in sterile 0.1 M PBS, and 100 μl was injected intraperitoneally into mice as a single dose per day, 5 x per week, starting at 2 months of age. At 6 months of age (after 4 months of metabolite supplementation), mice underwent behavioral testing as described in the “Behavior” section above. Brains and sera were collected, prepared and analyzed to determine effects on AD pathology as described in the sections above.

### Kinase ELISA

Total protein concentration of each brain homogenate was determined using the Rapid Gold BCA Protein Assay Kit (Pierce). Protein concentrations were standardized to 5 mg/mL using ice-cold 1 × DPBS. Samples were centrifuged at 20,000 × *g* for 20 min at 4 °C, and the supernatant was collected for subsequent analysis. Phosphorylated and total protein levels of ERK and GSK3β were measured using semi-quantitative ELISA kits, including the Mouse ERK ELISA Kit and the Mouse GSK3β (S9) ELISA Kit (RayBiotech), according to the manufacturer’s instructions.

### Fibril assay

Tau-441 (2N4R) P301S mutant monomers (10 μM; StressMarq) were combined with a solution containing 25 μM Thioflavin T (ThT) and 1 mM dithiothreitol (DTT) in DPBS. Monomer mixtures were aliquoted into a 96-well plate, and metabolites including thymidine, 2′-deoxyuridine, TMAO, 3IS, or phenol sulfate were added to the appropriate wells (final concentration in the wells: 200 μM). For cell-free tau fibrillization assays, metabolites were tested at a final concentration of 200 µM. This concentration was selected to approximate the upper range of metabolite exposure achieved in vivo following i.p. administration. A single standardized concentration was used across all metabolites to enable direct comparison of their intrinsic aggregation-promoting capacity, independent of differences in in vivo pharmacokinetics or baseline abundance. Prior in vitro studies have shown that metabolites such as TMAO can promote tau aggregation, but typically at highly supraphysiological concentrations (e.g., 200 mM–1 M)^29^. The present conditions were therefore chosen to assess aggregation under more physiologically relevant exposure levels. Well contents were mixed by gentle pipetting. ThT fluorescence was monitored using an excitation wavelength of 450 nm and an emission wavelength of 480 nm with a BioTek Synergy HT Microplate Reader using Gen5 software. Plates were sealed and placed in a shaking incubator (800 rpm) at 37 °C, and fluorescence readings were collected at 30 min intervals for 2 h. After the 2 h time point, heparin was added to each well to a final concentration of 0.11 mg/mL to induce aggregation. Plates were resealed and returned to the shaker, and fluorescence measurements continued to be collected at 30 min intervals for an additional 3 h. Wells containing Tau-441 (2N4R) P301S mutant fibrils (StressMarq) in place of monomers were included as positive controls. To summarize fibril formation, the area under the fluorescence-time curve (AUC) was calculated for each sample.

In a subset of technical replicates (e.g., two of three wells for thymidine and 2′-deoxyuridine), fluorescence increased rapidly following heparin addition and exceeded the dynamic range of the detector; these saturated timepoint measurements are represented as missing values and not included in the calculations. For 3IS, one intermediate time point was missing due to an error; the remaining measurements were used for analysis.

### Statistics and reproducibility

Mice used in the study were randomized to experimental groups. Sample sizes were powered according to numbers and levels of variation reported previously in Woerman et al. 2017^23^. For neuropathological analysis, experimenters were blinded of treatment groups when performing quantification for all immunohistochemistry experiments. For all figures. “n” indicates the number of independent replicates. Quantitative RT–PCR, immunofluorescence staining, and in vitro assays were replicated at least three times with similar results. Statistical analysis was performed using Prism (v10; GraphPad). Data were assessed for normal distribution to determine appropriate statistical testing and plotted in the figures as mean ± SEM. An outlier test using the ROUT method was performed for experiments that appeared to have visual outliers, and any identified outlier samples or animals were excluded from further analysis. Differences between the two treatment groups were assessed using an unpaired two-tailed Welch’s *t* test. Differences among > 2 groups with one variable were assessed using one-way ANOVA with a Dunnett’s post hoc test. Differences among > 2 groups with 2 variables were assessed using two-way ANOVA with Tukey’s post hoc test or Sidak’s correction as indicated in legends. Significant differences are indicated in the figures by *p < 0.05, **p < 0.01, ***p < 0.001, ****p < 0.0001. Nonsignificant differences are indicated in the figures with an “ns”.

### Reporting summary

Further information on research design is available in the Nature Portfolio Reporting Summary linked to this article.

## Supplementary information

Supplementary Information

Description of Additional Supplementary Files (PDF)

Supplementary Data 1

Supplementary Data 2

Supplementary Data 3

Supplementary Data 4

Supplementary Data 5

Supplementary Data 6

Supplementary Data 7

Supplementary Data 8

Supplementary Data 9

Supplementary Data 10

Reporting Summary

Transparent Peer Review file

## Source data

Supplementary Source Data

## Data Availability

All data are available in the article and its supplementary information files. The following data has also been deposited to the data repositories as indicated. Data from 16S rRNA gene sequencing, metagenomic profiling, and associated metadata are available online through the NCBI Sequence Read Archive (SRA) repository at https://www.ncbi.nlm.nih.gov/sra/PRJNA1338993. Metabolomic data are available online through Mendeley Data 10.17632/dvyvkrb59z.2. Source data are provided with this paper.
