# Supplementary material for: Select microbial metabolites promote tau aggregation in a murine tauopathy model
Source: Nat Commun. 2026 Jun 30;17:8161. doi: 10.1038/s41467-026-74775-6 (PMC13458332; doi:10.1038/s41467-026-74775-6)
Supplement: Supplementary file 1 [file 41467_2026_74775_MOESM13_ESM.pdf]

nature portfolio

Corresponding author(s): Sabeen A. Kazmi & Elaine Y. Hsiao

Last updated by author(s): 2026.05.26

Reporting Summary

Nature Portfolio wishes to improve the reproducibility of the work that we publish. This form provides structure for consistency and transparency in reporting. For further information on Nature Portfolio policies, see our Editorial Policies and the Editorial Policy Checklist.

Statistics

For all statistical analyses, confirm that the following items are present in the figure legend, table legend, main text, or Methods section.

n/a	Confirmed	
☐	☒	The exact sample size (n) for each experimental group/condition, given as a discrete number and unit of measurement
☐	☒	A statement on whether measurements were taken from distinct samples or whether the same sample was measured repeatedly
☐	☒	The statistical test(s) used AND whether they are one- or two-sided<br>*Only common tests should be described solely by name; describe more complex techniques in the Methods section.*
☐	☒	A description of all covariates tested
☐	☒	A description of any assumptions or corrections, such as tests of normality and adjustment for multiple comparisons
☐	☒	A full description of the statistical parameters including central tendency (e.g. means) or other basic estimates (e.g. regression coefficient) AND variation (e.g. standard deviation) or associated estimates of uncertainty (e.g. confidence intervals)
☐	☒	For null hypothesis testing, the test statistic (e.g. F, t, r) with confidence intervals, effect sizes, degrees of freedom and P value noted<br>*Give P values as exact values whenever suitable.*
☒	☐	For Bayesian analysis, information on the choice of priors and Markov chain Monte Carlo settings
☒	☐	For hierarchical and complex designs, identification of the appropriate level for tests and full reporting of outcomes
☐	☒	Estimates of effect sizes (e.g. Cohen's d, Pearson's r), indicating how they were calculated

Our web collection on statistics for biologists contains articles on many of the points above.

Software and code

Policy information about availability of computer code

Data collection	No software was used.
Data analysis	Open field and Barnes Maze behavior were analyzed using EthoVision XT (Noldus) software.<br>16S sequencing was analyzing using QIIME2 2025.4 (with DADA2, greengenes 2 database, vegan library v2.7.1, ANCOM-BC2 and logistf library); plots were visualized R v4.4.1 using ggplot2 v3.5.2 and ggpubr v0.6.1.<br>For metagenomics, raw sequence reads were processed using KneadData for quality filtering and removal of mouse-derived reads. MetaPhlAn4 was used to identify bacterial species present and compositional profiles . HUMAnN3 was used to annotate bacterial genes and determine functional roles and pathways. Bacterial gene abundances were aggregated into metabolic pathways using MetaCyc pathway classifications. Differential abundance of log-transformed KO genes or pathway counts was conducted using Maaslin3 v3.0.1 with fixed_formula = mouse sex + sampling timepoints and min_prevalence = 0.2. Plots were visualized R v4.4.1 using ggplot2 v3.5.2 and ggpubr v0.6.1.<br>For genomic adaptations, single nucleotide varients (SNVs) were identified by co-assembling metagenome-assembled genomes (MAGs) per mouse, followed by constructing a dereplicated MAG reference database with MIDAS v3.0.1 to assess species abundance, SNVs, and gene content. KEGG ids were obtained by running the corresponding amino acid sequence for the gene in the representative genome for that species through BlastKOALA. Analyses performed in Python and visualizations in R. Python v3.8.18 was used for all analyses, including generation of tables. Graphs were generated in R v4.4.1 using ggplot2 v3.5.2 and ggpubr v0.6.1.<br>For untargeted metabolomics, one-way ANOVA contrasts were performed in ArrayStudio on log transformed data. Principal components analysis plots were generated in R v4.4.1 using ggplot2 v3.5.111 and vegan v2.6.6.112. The Metaboanalyst 6.0 platform's metabolite set enrichment analysis (MSEA) was performed and analyzed for metabolite pathway enrichment using the Kyoto Encyclopedia of Genes and Genomes (KEGG). MSEA plots were generated in R v4.4.1 using ggplot2 v3.5.111. Metabolites/tau correlation heatmap was generated with R v4.4.1 using ggplot2 V3.5.2 and ggpubr v0.6.1.

Multiscale Embedded Gene Co-expression Network Analysis (MEGENA) was used to construct tissue-specific metabolite coexpression networks from mouse brain and serum metabolomics. Module eigengenes were calculated using the Weighted Gene Co-expression Network Analysis (WGCNA) moduleEigengenes. Plots were generated in R v.4.4.1 using ggplot2 v3.5.111.
Heatmap for the cell assay was generated with R v4.4.1 using pheatmap v1.0.12. All bar plots, line graphs, volcano plots were created and statistics were run in Prism v10.1.0 (Graphpad).

For manuscripts utilizing custom algorithms or software that are central to the research but not yet described in published literature, software must be made available to editors and reviewers. We strongly encourage code deposition in a community repository (e.g. GitHub). See the Nature Portfolio guidelines for submitting code & software for further information.

Data

Policy information about availability of data

All manuscripts must include a data availability statement. This statement should provide the following information, where applicable:

- Accession codes, unique identifiers, or web links for publicly available datasets
- A description of any restrictions on data availability
- For clinical datasets or third party data, please ensure that the statement adheres to our policy

16S rRNA gene sequencing and metagenomic data that support the findings are available in Supplementary Tables 1-5, and have been deposited to the NCBI Sequence Read Archive (SRA) repository at SRA: PRJNA1338993. Untargeted metabolomic data that support the findings are available in Supplementary Tables 6-7 and have been deposited to Mendeley data: https://doi: 10.17632/dvyvkrb59z.1.

Research involving human participants, their data, or biological material

Policy information about studies with human participants or human data. See also policy information about sex, gender (identity/presentation), and sexual orientation and race, ethnicity and racism.

Reporting on sex and gender

Use the terms sex (biological attribute) and gender (shaped by social and cultural circumstances) carefully in order to avoid confusing both terms. Indicate if findings apply to only one sex or gender; describe whether sex and gender were considered in study design; whether sex and/or gender was determined based on self-reporting or assigned and methods used.
Provide in the source data disaggregated sex and gender data, where this information has been collected, and if consent has been obtained for sharing of individual-level data; provide overall numbers in this Reporting Summary. Please state if this information has not been collected.
Report sex- and gender-based analyses where performed, justify reasons for lack of sex- and gender-based analysis.

Reporting on race, ethnicity, or other socially relevant groupings

Please specify the socially constructed or socially relevant categorization variable(s) used in your manuscript and explain why they were used. Please note that such variables should not be used as proxies for other socially constructed/relevant variables (for example, race or ethnicity should not be used as a proxy for socioeconomic status).
Provide clear definitions of the relevant terms used, how they were provided (by the participants/respondents, the researchers, or third parties), and the method(s) used to classify people into the different categories (e.g. self-report, census or administrative data, social media data, etc.)
Please provide details about how you controlled for confounding variables in your analyses.

Population characteristics

Describe the covariate-relevant population characteristics of the human research participants (e.g. age, genotypic information, past and current diagnosis and treatment categories). If you filled out the behavioural & social sciences study design questions and have nothing to add here, write "See above."

Recruitment

Describe how participants were recruited. Outline any potential self-selection bias or other biases that may be present and how these are likely to impact results.

Ethics oversight

Identify the organization(s) that approved the study protocol.

Note that full information on the approval of the study protocol must also be provided in the manuscript.

Field-specific reporting

Please select the one below that is the best fit for your research. If you are not sure, read the appropriate sections before making your selection.

[x] Life sciences [ ] Behavioural & social sciences [ ] Ecological, evolutionary & environmental sciences

For a reference copy of the document with all sections, see nature.com/documents/nr-reporting-summary-flat.pdf

Life sciences study design

All studies must disclose on these points even when the disclosure is negative.

Sample size

Sample sizes were based upon similar previously published study (Woerman et al. 2017 PMID 29059326).

Data exclusions

Samples were only excluded if they were identified as outliers using the ROUT method (Graphpad Prism).

Replication

Behavior and ELISA metrics were compiled from multiple experimental cohorts.
Cell assay data were complied from different passaged cells.

	Each mouse or passaged cells was treated as a biological replicate. 3 technical replicates (or 6 for the cell assay) were averaged for each biological replicate.<br>Fibril assay data were complied from three different technical replicates.<br>All attempts at replication were sucessful.
Randomization	All mice were randomly assigned to treatment groups.
Blinding	Experimenter was blinded to conditions when performing neuropatholgy analysis. Since mice were being treated during behavioral assessment, blinding was not possible. Blinding was not implemented for instrument-based assays due to automated data acquisition and analysis.

Reporting for specific materials, systems and methods

We require information from authors about some types of materials, experimental systems and methods used in many studies. Here, indicate whether each material, system or method listed is relevant to your study. If you are not sure if a list item applies to your research, read the appropriate section before selecting a response.

Materials & experimental systems

n/a	Involved in the study
☐	☒ Antibodies
☐	☒ Eukaryotic cell lines
☒	☐ Palaeontology and archaeology
☐	☒ Animals and other organisms
☒	☐ Clinical data
☒	☐ Dual use research of concern
☒	☐ Plants

Methods

n/a	Involved in the study
☒	☐ ChIP-seq
☐	☒ Flow cytometry
☒	☐ MRI-based neuroimaging

Antibodies

Antibodies used	Phospho-Tau (Ser202, Thr205) Biotinylated AT8 (1:200; ThermoFisher, cat# MN1020B), anti-Iba-1 (rabbit; 1:500; Wako, cat# 019-19741) and GFAP (chicken; 1:500; ThermoFisher, cat# PA1-10004)Alexa Fluor 488 (Streptavidin; 1:1000; ThermoFisher, cat# S11223), Alexa Fluor 750 (goat anti-rabbit; 1:1000; ThermoFisher, cat# A21039), and Alexa Fluor 555 (goat anti-chicken; 1:1000; ThermoFisher, cat# A32932). All antibodies have been verified by Thermofisher or Fujifilm (per their website) and have been cited in numerous papers.
Validation	Both antibodies come with a certificate of analysis and have been throughly cited in published research articles.

Eukaryotic cell lines

Policy information about cell lines and Sex and Gender in Research

Cell line source(s)	Tau RD P301S FRET Biosensor cells (ATCC, cat# CRL-3275) Made from transducing HEK293T (female) cells with lentivirus vectors.
Authentication	Deposited to ATCC by M. Diamond, University of Texas Southwestern Medical Center. Authenticated by ATCC using STR profiling.
Mycoplasma contamination	no contamination
Commonly misidentified lines<br>(See ICLAC register)	None

Animals and other research organisms

Policy information about studies involving animals; ARRIVE guidelines recommended for reporting animal research, and Sex and Gender in Research

Laboratory animals	Mice used in this study included 5xFAD, 3xTg, C57BL/6J, and hTau.P301S. All strains were on a C57BL/6J background. 5xFAD, 3xTg, and C57BL/6J mice were obtained from Jackson Laboratory, and hTau.P301S mice were provided by the Goedert Lab and bred at the UCLA animal facility. Animals were maintained in autoclaved cages under standard housing conditions on a 12 h light/12 h dark cycle with ad libitum access to chow and water. All procedures were approved by the UCLA Institutional Animal Care and Use Committee. Experiments began at 4 weeks of age. Fecal samples were collected monthly starting at 2 months of age. Behavioral testing took place at 6 months.
Wild animals	This study did not involve wild animals.
Reporting on sex	Equal number of males and females were for all experiments, but sex was not included during analysis.

Field-collected samples | This study did not involve field-collected samples.

Ethics oversight | All experiments were performed in accordance with the NIH Guide for the Care and Use of Laboratory Animals using protocols approved by the Institutional Animal Care and Use Committee at UCLA.

Note that full information on the approval of the study protocol must also be provided in the manuscript.

Plants

Seed stocks | *Report on the source of all seed stocks or other plant material used. If applicable, state the seed stock centre and catalogue number. If plant specimens were collected from the field, describe the collection location, date and sampling procedures.*

Novel plant genotypes | *Describe the methods by which all novel plant genotypes were produced. This includes those generated by transgenic approaches, gene editing, chemical/radiation-based mutagenesis and hybridization. For transgenic lines, describe the transformation method, the number of independent lines analyzed and the generation upon which experiments were performed. For gene-edited lines, describe the editor used, the endogenous sequence targeted for editing, the targeting guide RNA sequence (if applicable) and how the editor was applied.*

Authentication | *Describe any authentication procedures for each seed stock used or novel genotype generated. Describe any experiments used to assess the effect of a mutation and, where applicable, how potential secondary effects (e.g. second site T-DNA insertions, mosiacism, off-target gene editing) were examined.*

Flow Cytometry

Plots

Confirm that:

- [x] The axis labels state the marker and fluorochrome used (e.g. CD4-FITC).
- [x] The axis scales are clearly visible. Include numbers along axes only for bottom left plot of group (a 'group' is an analysis of identical markers).
- [x] All plots are contour plots with outliers or pseudocolor plots.
- [x] A numerical value for number of cells or percentage (with statistics) is provided.

Methodology

Sample preparation | Cell assay was conducted as described in the Methods section. Cells were trypsinized, resuspended in DPBS, and analyzed for CFP, FRET, and YFP fluorescence using specific laser excitations and emission filters (CFP/FRET ex405nm em440/50nm and 512/50nm, YPF ex488nm em 525/50nm).

Instrument | Cells were analyzed using he Attune NxT Flow Cytometer (ThermoFIsher) with the CytKick Autosampler (ThermoFisher).

Software | Further analysis was performed using FlowJo v10.

Cell population abundance | FRET was quantified using a bivariate FRET vs. CFP plot with a triangular gate set based on PBS-only controls to exclude FRET-negative cells. For each experiment, 20,000 cells per well were analyzed. Data collected included raw counts, percentage of FRET-positive cells, and geometric mean fluorescence intensity. Integrated FRET density was calculated by multiplying the percentage of FRET-positive cells by the geometric mean fluorescence intensity.

Gating strategy | Gating strategy is provided Supplementary Fig. 7a).

- [x] Tick this box to confirm that a figure exemplifying the gating strategy is provided in the Supplementary Information.
