# Supplementary material for: Select microbial metabolites promote tau aggregation in a murine tauopathy model
Source: Nat Commun. 2026 Jun 30;17:8161. doi: 10.1038/s41467-026-74775-6 (PMC13458332; doi:10.1038/s41467-026-74775-6)
Supplement: Supplementary file 2 [file 41467_2026_74775_MOESM14_ESM.pdf]

nature portfolio

Peer Review File

Select microbial metabolites promote tau aggregation in a murine tauopathy model

Corresponding Author: Professor Elaine (Y) Hsiao

This manuscript has been previously reviewed at another journal. This document only contains information relating to versions considered at Nature Communications.

This file contains all reviewer reports in order by version, followed by all author rebuttals in order by version.

Version 0:

Reviewer comments:

Reviewer #3

(Remarks to the Author)
The manuscript has been thoroughly revised, and the authors have provided appropriate responses. Regarding tau pathology in hTau.P301S mice, immunohistochemical analysis using the phosphorylated tau antibody AT8 demonstrated enhanced staining in the pons of 6-month-old transgenic mice. This reviewer agrees that these mice exhibit pronounced tau pathology in this region. However, it would be informative to determine whether a similar acceleration of tau pathology occurs in the hippocampus and/or cerebral cortex following ABX treatment in transgenic mice. If such effects are observed, the statement in lines 188–189 referring to “broader circuit-level dysfunction in hippocampal-dependent spatial memory” could be refined by incorporating reference to “pathological tau accumulation.” The reviewer would also like clarification on whether immunohistochemical analyses were performed in hippocampal and cortical regions in these experiments.

Reviewer #4

(Remarks to the Author)
This review comment was prepared by Dong-oh Seo at the Medical College of Georgia at Augusta University as the fourth reviewer.

Recent accumulating data suggest that the gut microbiome regulates the progression of AD. However, the detailed mechanisms by which the gut microbiota influence the disease remain under scrutiny. Gut microbiota generate various metabolites, which may directly contribute to brain pathology or indirectly regulate AD pathologies via peripheral immunity and metabolism.

In this manuscript, Sabeen A. Kazmi and colleagues identified a unique set of microbiota-derived metabolites that are more likely to directly contribute to the progression of tau pathology. They used various techniques, including antibiotic administration in Thy1-P301S hTau mice, microbiome and metabolomic approaches, and biosensor assays. This is a very interesting study and has several important implications for the field. I believe that the authors have responded well to the previous reviewer’s comments.

As the author of a previous report showing that the antibiotics treatments to p301s-apoe3 mice reduce tau-mediated neurodegeneration (PMID: 36634180), I find it intriguing (and encouraging) to see the facilitating effect of antibiotic treatment in hTau p301s mice. Regarding the inconsistency, rather than discouraging the reporting of this study, these findings provide an important and balanced perspective for the field, as they reflect the dynamic involvement of the gut microbiome in disease progression. This is also a good opportunity to clarify that, similar to these authors, I used antibiotics as a tool to “perturb” the gut microbiome to investigate its role in pathology development, rather than to argue that antibiotics are clinically beneficial or harmful: can be but not necessarily.

Unlike germ-free approaches, in which animals are subjected to lifelong and complete microbial absence, the outcomes of antibiotic treatment can vary depending on which subsets of microbes are present or replenished, when treatment is administered, how long it is, and which stages of the disease are evaluated. While future studies will require more

systematic comparisons, this study provides an important starting point and is highly thought-provoking.

One minor point I would suggest to add is that it would be helpful to provide more details about which portion of the hindbrain was measured for pTau levels: was it the entire hindbrain or a specific neural structure? This would also be helpful to speculate on why there was an impairment in the spatial memory test.

Open Access This Peer Review File is licensed under a Creative Commons Attribution 4.0 International License, which permits use, sharing, adaptation, distribution and reproduction in any medium or format, as long as you give appropriate credit to the original author(s) and the source, provide a link to the Creative Commons license, and indicate if changes were made.
In cases where reviewers are anonymous, credit should be given to 'Anonymous Referee' and the source.
The images or other third party material in this Peer Review File are included in the article's Creative Commons license, unless indicated otherwise in a credit line to the material. If material is not included in the article's Creative Commons license and your intended use is not permitted by statutory regulation or exceeds the permitted use, you will need to obtain permission directly from the copyright holder.
To view a copy of this license, visit https://creativecommons.org/licenses/by/4.0/

We thank the Reviewers for their additional thoughtful comments and suggestions. We have addressed the remaining points through minor textual revisions, clarification of experimental details, and inclusion of supplementary data as described below.

Reviewer #3:

The manuscript has been thoroughly revised, and the authors have provided appropriate responses. Regarding tau pathology in hTau.P301S mice, immunohistochemical analysis using the phosphorylated tau antibody AT8 demonstrated enhanced staining in the pons of 6-month-old transgenic mice. This reviewer agrees that these mice exhibit pronounced tau pathology in this region. However, it would be informative to determine whether a similar acceleration of tau pathology occurs in the hippocampus and/or cerebral cortex following ABX treatment in transgenic mice. If such effects are observed, the statement in lines 188–189 referring to “broader circuit-level dysfunction in hippocampal-dependent spatial memory” could be refined by incorporating reference to “pathological tau accumulation.” The reviewer would also like clarification on whether immunohistochemical analyses were performed in hippocampal and cortical regions in these experiments.

We thank the reviewer for this helpful suggestion. In response, we quantified AT8-positive tau pathology in the hippocampus and entorhinal cortex of ABX-treated and control hTau.P301S mice. In contrast to the increased AT8 immunoreactivity observed in the pons, we did not detect significant differences in phosphorylated tau burden in either the hippocampus or entorhinal cortex at this disease stage.

We agree that these findings warrant a more conservative interpretation of the behavioral phenotype. Accordingly, we have revised the text in lines 186–188 to avoid directly linking the observed Barnes maze deficits to pathological tau accumulation within hippocampal-associated memory circuits. We have also included the hippocampal and entorhinal cortex analyses as supplementary figures.

Revised in the manuscript as lines 186-188: “Although tau pathology in this model was most prominent in the pons and medulla, ABX-treated hTau mice exhibited increased latency to locate the escape hole in the Barnes maze, indicating impaired spatial memory performance.”

New figures added to the manuscript:

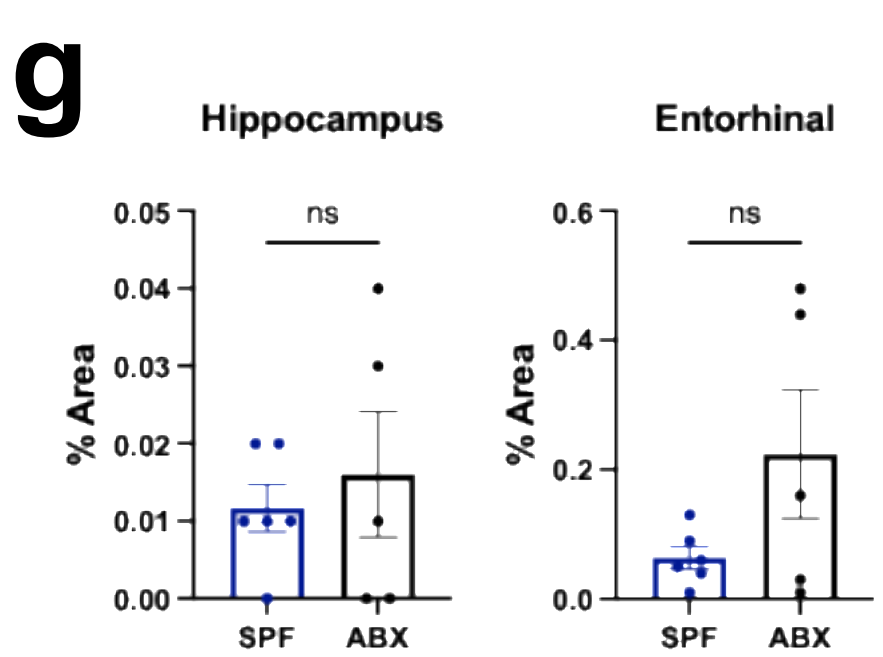

Extended Data Fig. 4g (g) Quantification of tau (AT8) pathology in the hippocampus and entorhinal cortex of 6-month-old brains of ABX (n=5) and SPF (n=6) hTau mice (two-tailed Welch’s t test).

Reviewer #4:

This review comment was prepared by Dong-oh Seo at the Medical College of Georgia at Augusta University as the fourth reviewer.

Recent accumulating data suggest that the gut microbiome regulates the progression of AD. However, the detailed mechanisms by which the gut microbiota influence the disease remain under scrutiny. Gut microbiota generate various metabolites, which may directly contribute to brain pathology or indirectly regulate AD pathologies via peripheral immunity and metabolism.

In this manuscript, Sabeen A. Kazmi and colleagues identified a unique set of microbiota-derived metabolites that are more likely to directly contribute to the progression of tau pathology. They used various techniques, including antibiotic administration in Thy1-P301S hTau mice, microbiome and metabolomic approaches, and biosensor assays. This is a very interesting study and has several important implications for the field. I believe that the authors have responded well to the previous reviewer's comments.

As the author of a previous report showing that the antibiotics treatments to p301s-apoe3 mice reduce tau-mediated neurodegeneration (PMID: 36634180), I find it intriguing (and encouraging) to see the facilitating effect of antibiotic treatment in hTau p301s mice. Regarding the inconsistency, rather than discouraging the reporting of this study, these findings provide an important and balanced perspective for the field, as they reflect the dynamic involvement of the gut microbiome in disease progression. This is also a good opportunity to clarify that, similar to these authors, I used antibiotics as a tool to "perturb" the gut microbiome to investigate its role in pathology development, rather than to argue that antibiotics are clinically beneficial or harmful: can be but not necessarily.

Unlike germ-free approaches, in which animals are subjected to lifelong and complete microbial absence, the outcomes of antibiotic treatment can vary depending on which subsets of microbes are present or replenished, when treatment is administered, how long it is, and which stages of the disease are evaluated. While future studies will require more systematic comparisons, this study provides an important starting point and is highly thought-provoking.

One minor point I would suggest to add is that it would be helpful to provide more details about which portion of the hindbrain was measured for pTau levels: was it the entire hindbrain or a specific neural structure? This would also be helpful to speculate on why there was an impairment in the spatial memory test.

We thank the reviewer for the thoughtful comments and for placing our findings in the broader context of microbiome perturbation studies in tauopathy models. We appreciate the reviewer's perspective regarding the use of antibiotics as an experimental tool to perturb the gut microbiome rather than as an inherently beneficial or detrimental intervention.

We also thank the reviewer for the suggestion regarding clarification of the hindbrain region analyzed for phosphorylated tau. In these experiments, immunohistochemical quantification was performed specifically in the pons and medulla rather than across the entire hindbrain. We will clarify this explicitly in the Results and Methods section.

Revised in the manuscript as lines 186-188: "Although tau pathology in this model was most prominent in the pons and medulla, ABX-treated hTau mice exhibited increased latency to locate the escape hole in the Barnes maze, indicating impaired spatial memory performance."

Revised in the manuscript as lines 730-733: "The percent area of antibody-positive pixels was quantified in the hippocampus, entorhinal cortex and hindbrain. For the hindbrain analysis, the pons and medulla were combined and analyzed as a single region of interest."
