# Supplementary material for: Select microbial metabolites promote tau aggregation in a murine tauopathy model
Source: Nat Commun. 2026 Jun 30;17:8161. doi: 10.1038/s41467-026-74775-6 (PMC13458332; doi:10.1038/s41467-026-74775-6)
Supplement: Supplementary file 3 [file 41467_2026_74775_MOESM1_ESM.pdf]

Supplementary Information for:

Select microbial metabolites promote tau aggregation in a murine tauopathy model

Sabeen A. Kazmi*, Franciscus Chandra, Michael Wasney, Jenny Cheng, Gregory R. Lum, Malvika Iyer, Daria Di Blasi, Adriana N. Espinoza, Arlene Lopez-Romero, Xia Yang, Nandita Garud and Elaine Y. Hsiao*

*Correspondence to: sak14@g.ucla.edu, ehsiao@g.ucla.edu

This PDF file includes:

Supplementary Figures 1-9

(Raw input data for each graph of all figures are provided in the Source Data file)

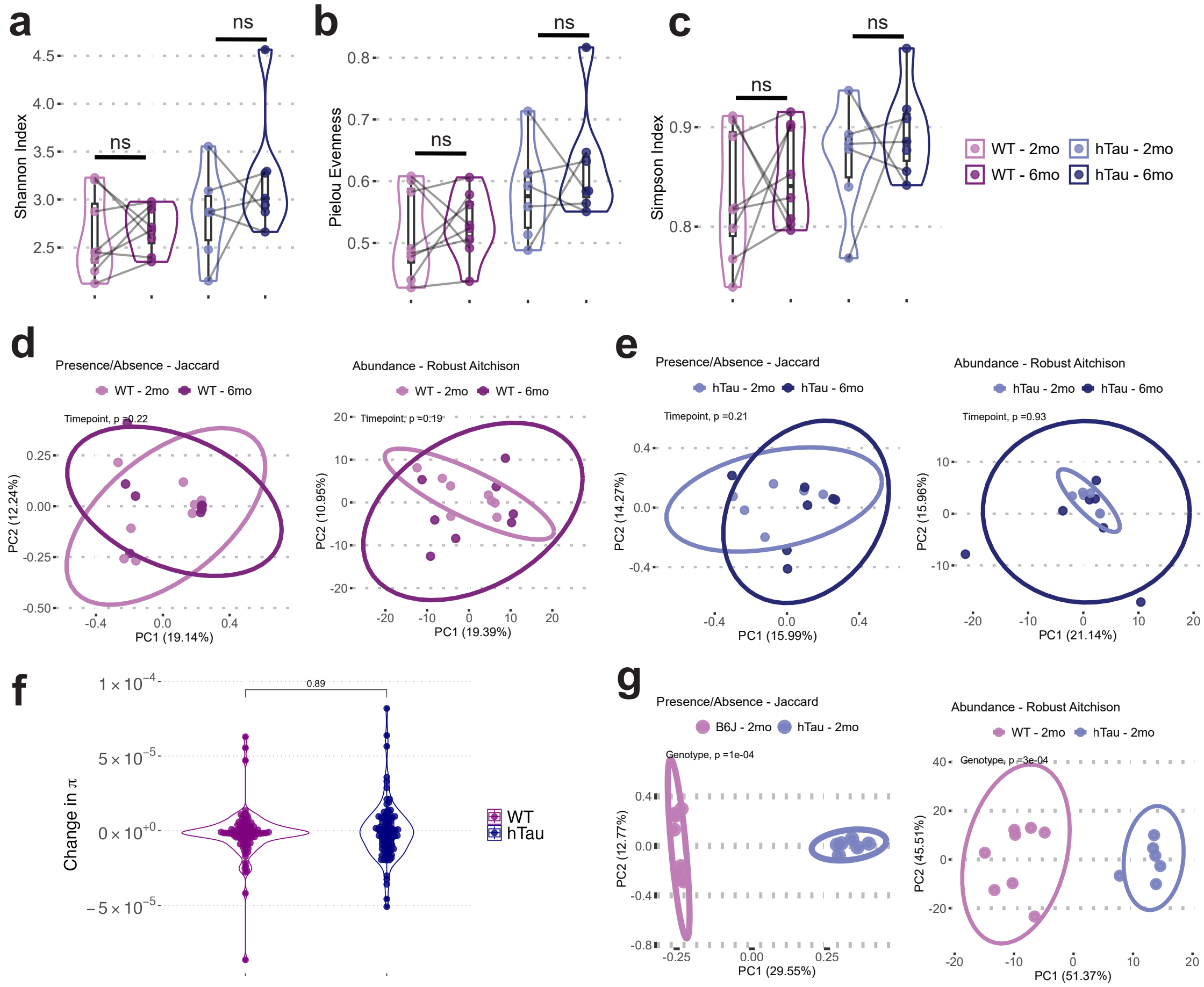

Fig. S1 | There are no significant differences in other microbial diversity measures over the course of disease in hTau.P301S mice

(a-c) Measures of alpha diversity at 2 months (hTau n=6; WT n=8) and 6 months (hTau n=7; WT n=8) of age. (Wilcoxon signed rank test). (a) Shannon's Diversity Index (b) Pielou's Evenness Index (c) Simpson Index (d) Principal coordinates analysis of Jaccard and Robust Aitchison distances from 16S rRNA gene sequencing of WT SPF mice at 2 months (n=8) and 6 months (n=8) of age. (PERMANOVA). (d) Principal coordinates analysis of Jaccard and Robust Aitchison distances from 16S rRNA gene sequencing of hTau SPF mice at 2 months (n=6) and 6 months (n=7) of age. (PERMANOVA). (f) Change in π, or nucleotide diversity within a population, between 2 and 6 months was calculated for WT and hTau mice (n=45) detected at both timepoints within at least one WT (n=7) and hTau (n=8) mouse. Each dot represents the change in π for a particular species. The median change in π was not significantly different between genotype (Wilcoxon rank sum test). (g) Principal coordinates analysis of Jaccard and Robust Aitchison distances from 16S rRNA gene sequencing of WT SPF mice at 2 months (n=8) and hTau mice at 2 months (n=6) of age. (PERMANOVA). ns=not statistically significant.

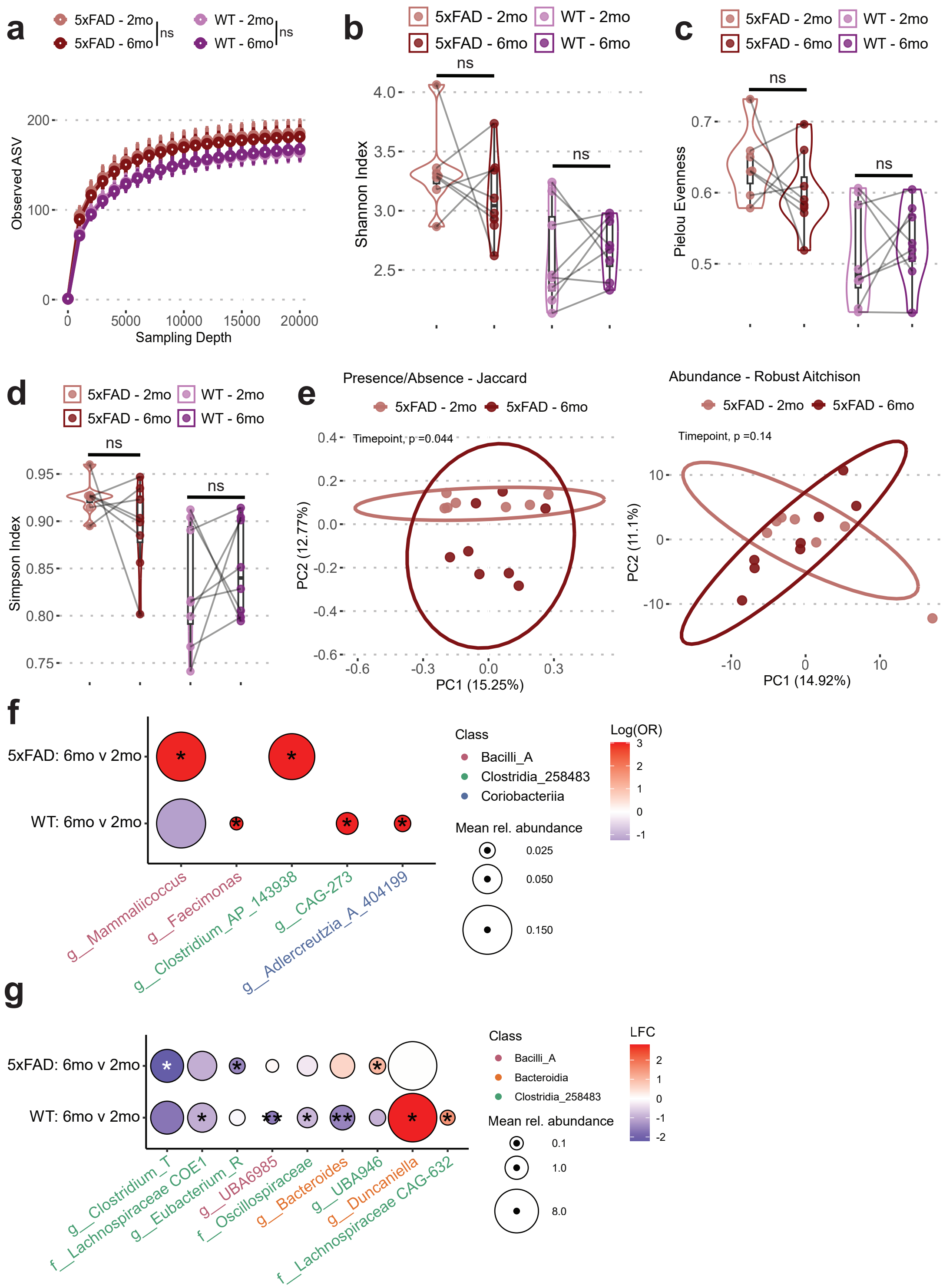

Fig. S2 | Key microbiota alterations observed in hTau.P301S mice are not seen in 5xFAD mice

(a) Rarefaction curve of sequenced ASVs from 5xFAD and WT SPF mouse fecal microbiome samples collected at 2 months (5xFAD n=6; WT n=8) and 6 months (5xFAD n=8; WT n=8; Kruskal-Wallis). All data for the WT SPF control are same as in main Figure 1. (b-d) Measures of alpha diversity at 2 months (5xFAD n=8; WT n=8) and 6 months (5xFAD n=8; WT n=8) of age

(Wilcoxon signed rank test). (b) Shannon's Diversity Index (c) Pielou's Evenness Index (d) Simpson Index (e) Principal coordinate analysis of Jaccard and Robust Aitchison distances from 16S rRNA gene sequencing of 5xFAD SPF mice at 2 months (n=8) and 6 months (n=8) of age. (PERMANOVA). (f) Differential presence/absence of significant microbes by logistic regression ($p<0.05$) at 6 months (5xFAD n=8; WT n=8) compared to 2 months (5xFAD n=8; WT) in 5xFAD and WT mice. (g) Differential abundance of significant microbes by ANCOM-BC2 testing ($p<0.05$) at 6 months (5xFAD n=8; WT n=8) compared to 2 months (5xFAD n=8; WT) in 5xFAD and WT mice. OR = odds ratio. LFC = log-fold change. Results in rarefaction plot are expressed as mean ± SEM. *$p<0.05$, **$p<0.01$, ns=not statistically significant.

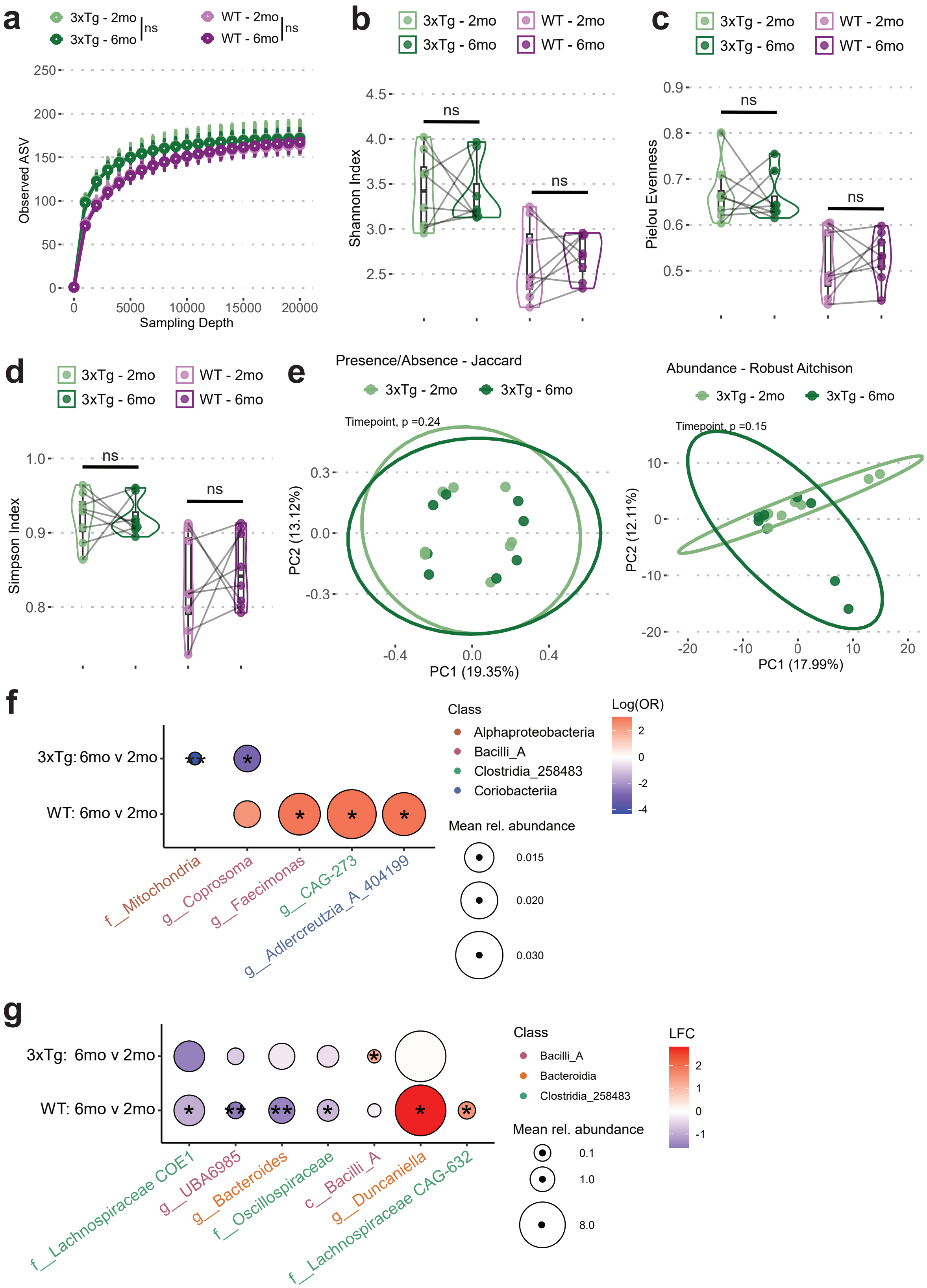
a
3xTg - 2mo
3xTg - 6mo
ns
WT - 2mo
WT - 6mo
ns
Observed ASV
Sampling Depth
b
Shannon Index
ns
ns
c
Pielou Evenness
ns
ns
d
Simpson Index
ns
ns
e
Presence/Absence - Jaccard
Timepoint, p =0.24
PC1 (19.35%)
PC2 (13.12%)
Abundance - Robust Aitchison
Timepoint, p =0.15
PC1 (17.99%)
PC2 (12.11%)
f
3xTg: 6mo v 2mo
WT: 6mo v 2mo
f__Mitochondria
g__Coprosoma
g__Faecimonas
g__CAG-273
g__Adlercreutzia_A_404199
Class
Alphaproteobacteria
Bacilli_A
Clostridia_258483
Coriobacteriia
Log(OR)
Mean rel. abundance
0.015
0.020
0.030
g
3xTg: 6mo v 2mo
WT: 6mo v 2mo
f__Lachnospiraceae COE1
g__UBA6985
g__Bacteroides
f__Oscillospiraceae
c__Bacilli_A
g__Duncaniella
f__Lachnospiraceae CAG-632
Class
Bacilli_A
Bacteroidia
Clostridia_258483
LFC
Mean rel. abundance
0.1
1.0
8.0

Fig. S3 | Key microbiota alterations observed in hTau.P301S mice are not seen in 3xTg mice (a) Rarefaction curve of sequenced ASVs from 3xTg and WT SPF mouse fecal microbiome samples collected at 2 months (3xTg n=6; WT n=8) and 6 months (3xTg n=8; WT n=8; Kruskal-Wallis). All data for the WT SPF control are same as in main Figure 1. (b-d) Measures of alpha diversity at 2 months (3xTg n=8; WT n=8) and 6 months (3xTg n=8; WT n=8) of age. (Wilcoxon signed rank test). (b) Shannon's Diversity Index (c) Pielou's Evenness Index (d) Simpson Index (e) Principal coordinate analysis of Jaccard and Robust Aitchison distances from 16s rRNA gene sequencing of 3xTg SPF mice at 2 months (n=8) and 6 months (n=8) of age. (PERMANOVA). (f) Differential presence/absence of significant microbes by logistic regression testing ($p<0.05$) at 6 months (3xTg n=8; WT n=8) compared to 2 months (3xTg n=8; WT n=8) in 3xTg and WT mice. (g) Differential abundance of significant microbes by ANCOM-BC2 testing ($p<0.05$) at 6 months (3xTg n=8; WT n=8) compared to 2 months (3xTg n=8; WT n=8) in 3xTg and WT mice. OR = odds ratio. LFC = log-fold change. Results in rarefaction plot are expressed as mean ± SEM. *$p<0.05$, **$p<0.01$, ns=not statistically significant.

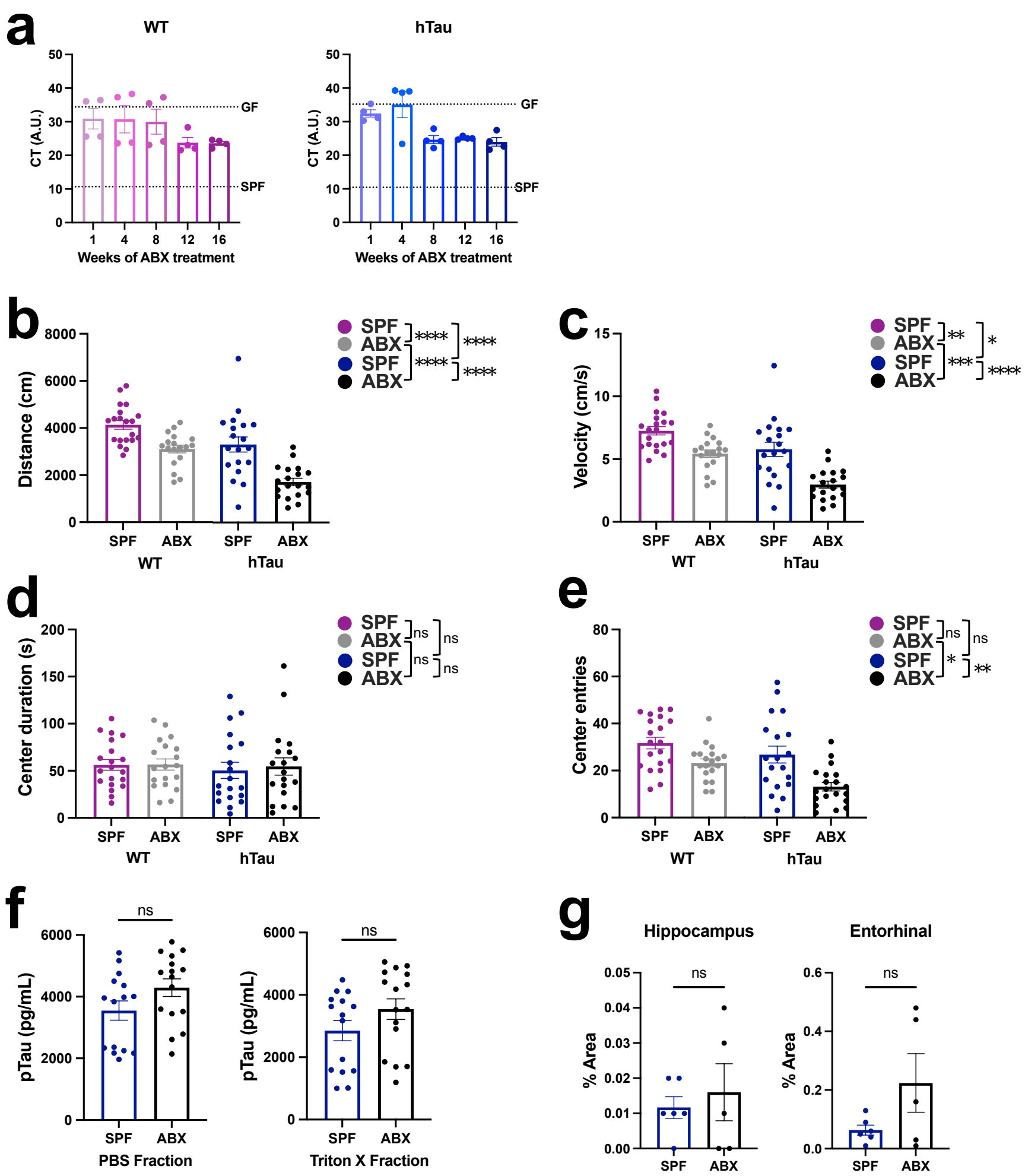

Fig. S4 | Validation of microbiota depletion with no genotype differences in open-field behavior, and characterized tau pathology in hTau brains

(a) Fecal bacterial load by 16S rRNA gene qPCR in ABX-treated WT and hTau mice (WT ABX n=4; hTau ABX n=4). (b-e) Open field test results for WT and hTau mice (WT ABX n=19, SPF n=20; hTau ABX n=20, SPF n=19; two-way ANOVA with Tukey's test). (b) Distance traveled (all p values=<0.0001 (c) Velocity (WT ABX/SPF p=0.0073; hTau ABX/SPF p=<0.0001; WT ABX/hTau ABX p=0.0002; WT SPF/hTau SPF p=0.0303) (d) Time spent in the center (e) Number of entries to the center (hTau ABX/SPF p=0.0013; WT ABX/hTau ABX p=0.0233) (f) Quantification of ptau in the PBS or triton X-100 fractions of 6-month-old brains of ABX (n=16) and SPF (n=15) hTau mice (two-tailed Welch's t test). (g) Quantification of tau (AT8) pathology in the hippocampus and entorhinal cortex of 6-month-old brains of ABX (n=5) and SPF (n=6) hTau mice (two-tailed Welch's t test). Results are expressed as mean ± SEM. **p<0.01, ***p<0.001, ns=not statistically significant.

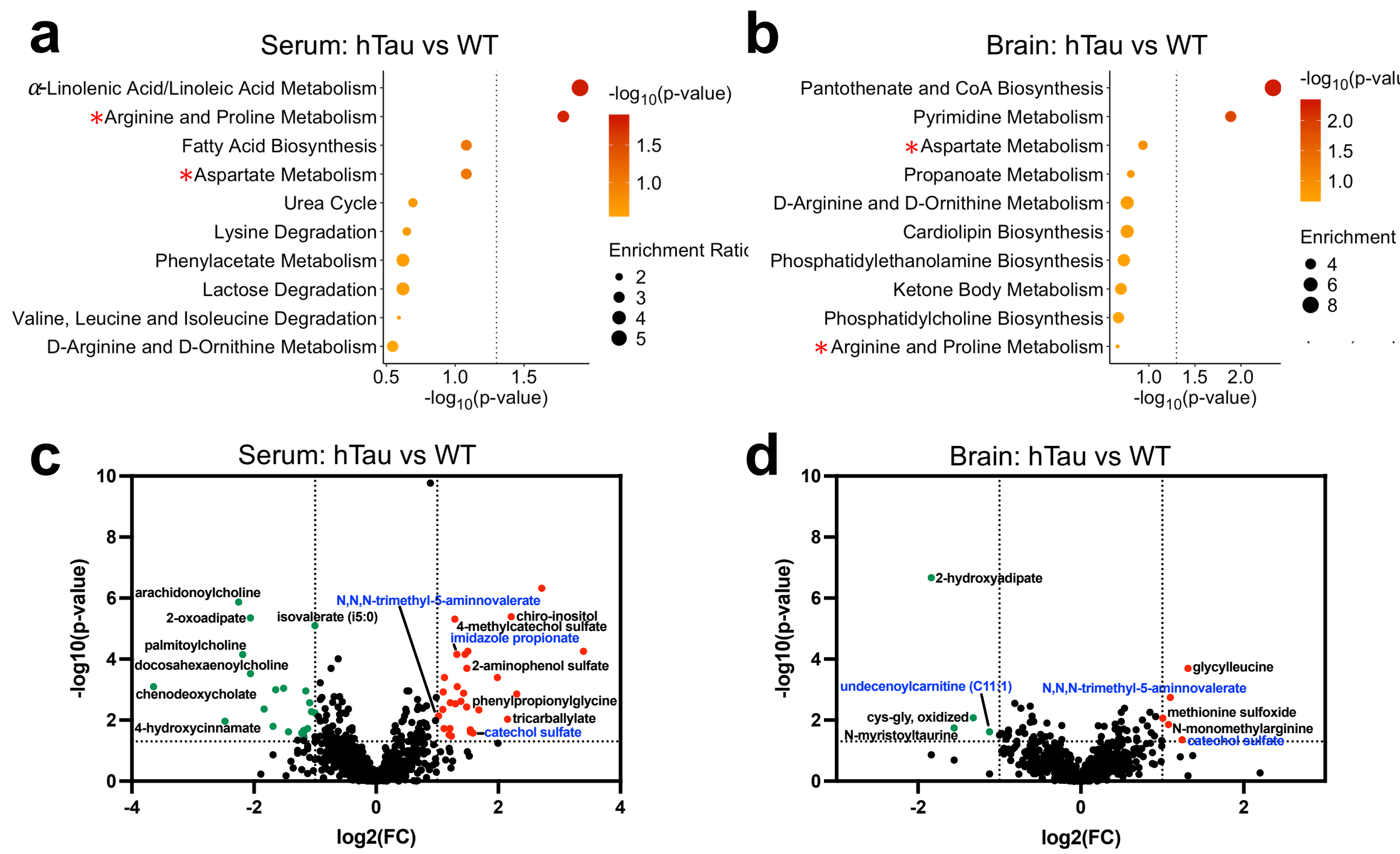

Fig. S5 | The gut microbiome regulates select metabolites in the serum and brain of hTau.P301S mice relative to WT controls

(a) Top 10 enriched Small Molecule Pathway Database pathways (SMPDB) for serum metabolites WT (n=6) compared to hTau (n=6) mice. ($p<0.05$, one-way ANOVA). Asterisks for indicate pathways that are significant for both serum and brain. (b) Top 10 enriched Small Molecule Pathway Database pathways (SMPDB)) for brain metabolites in WT (n=6) compared to hTau (n=6) mice. ($p<0.05$, one-way ANOVA). Asterisks for indicate pathways that are significant for both serum and brain. (c) Volcano plot highlighting high fold-change serum metabolites from hTau mice compared to WT controls. Metabolites candidates for in vitro testing highlighted in blue ($p<0.05$, one-way ANOVA; hTau n=6; WT n=6). (d) Volcano plot highlighting high fold-change brain metabolites from hTau compared to WT controls. Metabolites candidates for in vitro testing highlighted in blue ($p<0.05$, one-way ANOVA; hTau n=6; WT n=6).

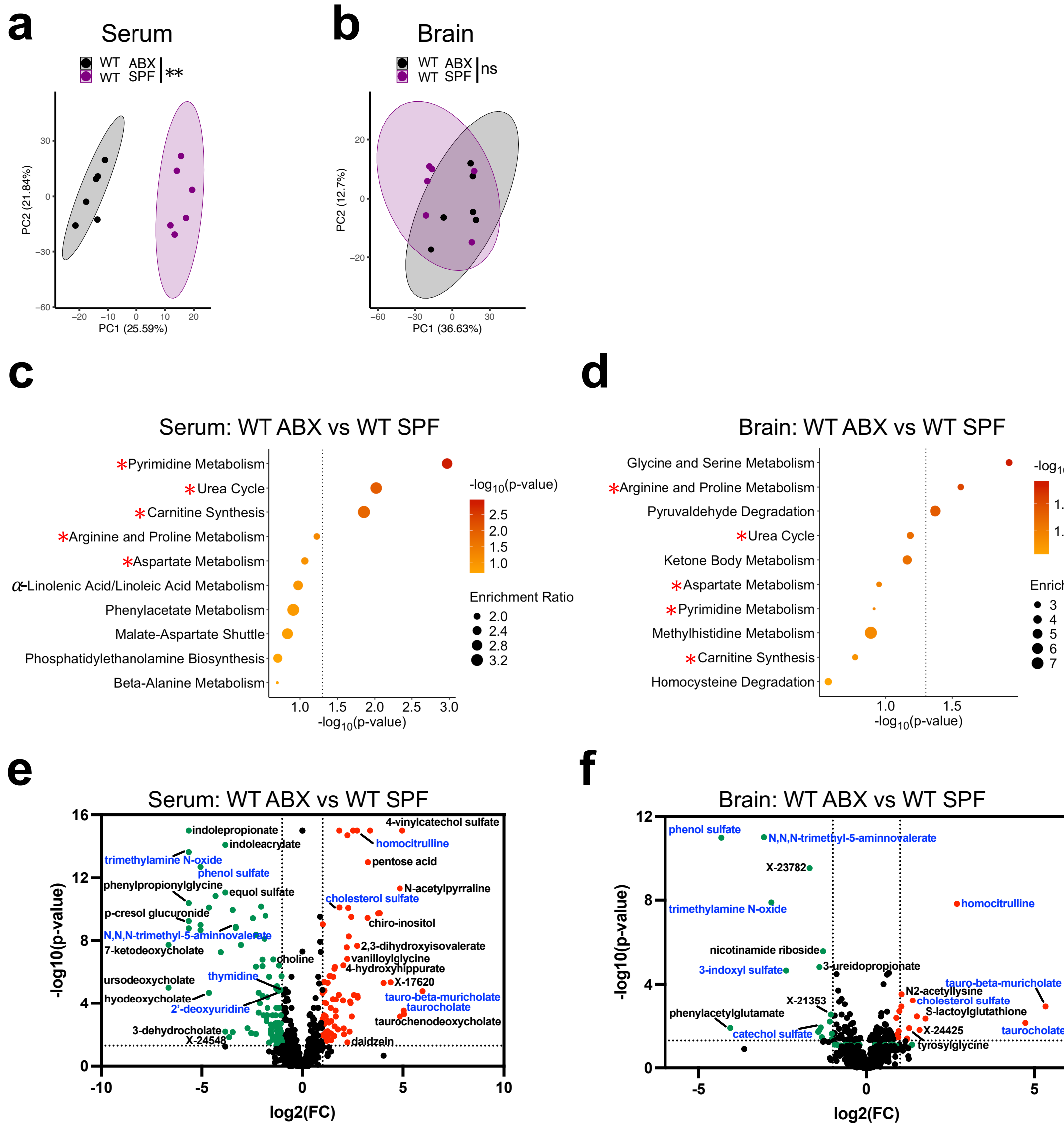

Fig. S6 | The gut microbiome regulates select metabolites in the serum and brain of WT mice (a) Principal component analysis of serum untargeted metabolomics data from SPF WT mice (n=6) compared to WT mice treated with ABX (n=6; PERMANOVA; p=0.002). (b) Principal component analysis of brain untargeted metabolomics from SPF WT mice (n=6) compared to WT mice treated with ABX (n=6; PERMANOVA). (c) Top 10 enriched Small Molecule Pathway Database pathways (SMPDB) for serum metabolites in ABX-treated (n=6) compared to SPF (n=6) WT mice (p<0.05, one-way ANOVA). Asterisks for indicate pathways that are significant for both serum and brain. (d) Top 10 enriched Small Molecule Pathway Database pathways (SMPDB)) for brain metabolites in ABX-treated (n=6) compared to SPF (n=6) WT mice (p<0.05, one-way ANOVA). Asterisks for indicate pathways that are significant for both serum and brain. (e) Volcano plot highlighting high fold-change serum metabolites from WT mice significantly altered by ABX treatment. Metabolites candidates for in vitro testing highlighted in bold (p<0.05, one-way ANOVA; ABX n=6; SPF n=6). (f) Volcano plot highlighting high fold-change brain metabolites from hTau mice significantly altered by ABX treatment. Metabolites candidates for in vitro testing

highlighted in blue ($p<0.05$, one-way ANOVA; ABX n=6; SPF n=6). **$p<0.01$, ns=not statistically significant.

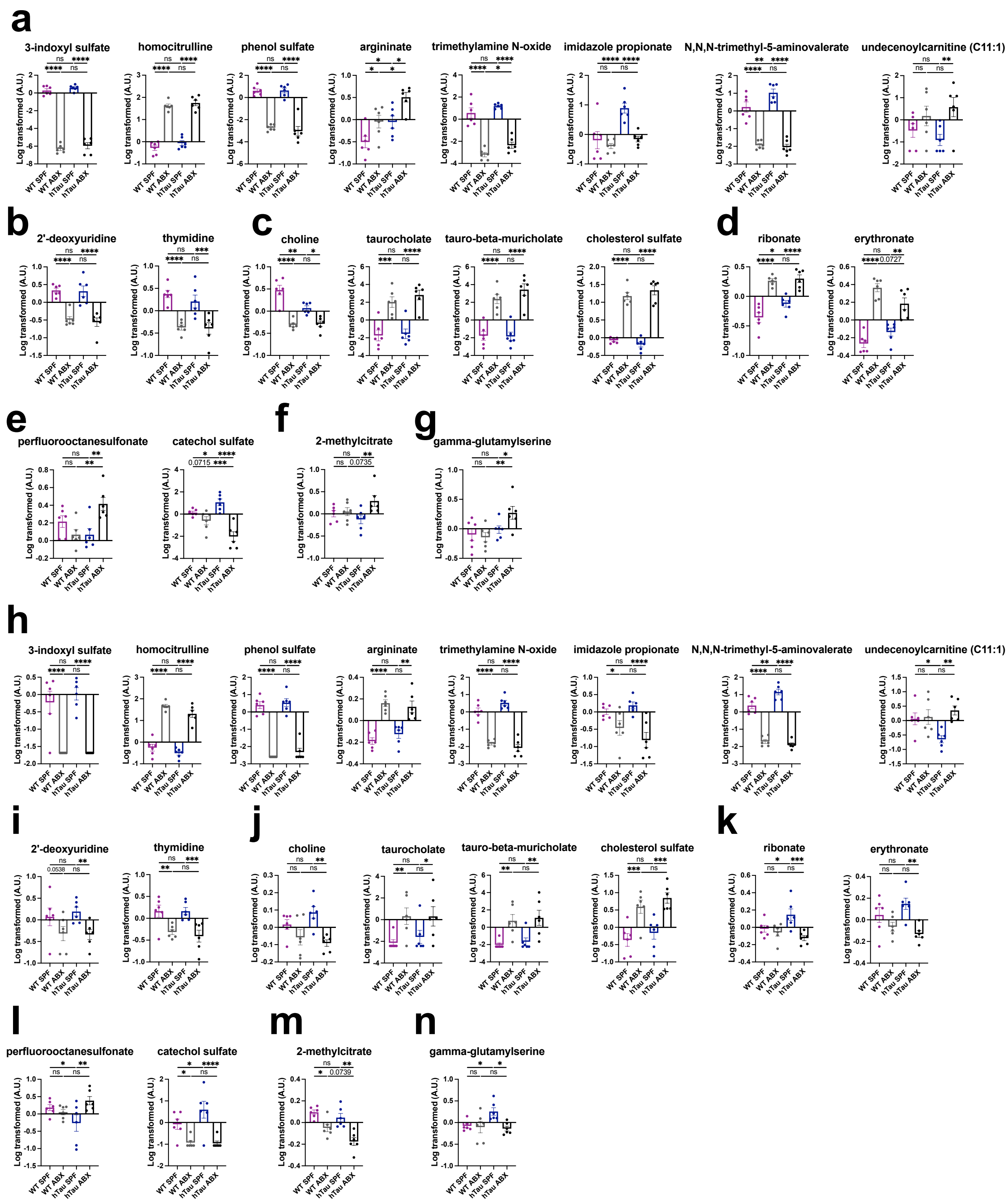

Fig. S7 | 20 select microbially modulated metabolites in the serum and brain that correlate with tau aggregation in hTau.P301S mice

(a-g) Relative concentrations of metabolites in the serum (one-way ANOVA; n=6 per group). Exact p values are provided in the Source Data. (a) amino acid metabolites (b) nucleotide

metabolites (c) lipid metabolites (d) carbohydrate metabolites (e) xenobiotic metabolites (f) energy metabolites (g) peptide metabolites. (h-n) Relative concentrations of metabolites in the brain (one-way ANOVA; n=6 per group). Exact p values are provided in the Source Data. (h) amino acid metabolites (i) nucleotide metabolites (j) lipid metabolites (k) carbohydrate metabolites (l) xenobiotic metabolites (m) energy metabolites (n) peptide metabolites. Values reflect natural log of normalized peak area per batch. Results are expressed as mean ± SEM. *$p<0.05$, **$p<0.01$, ***$p<0.001$, ****$p<0.0001$, ns=not statistically significant.

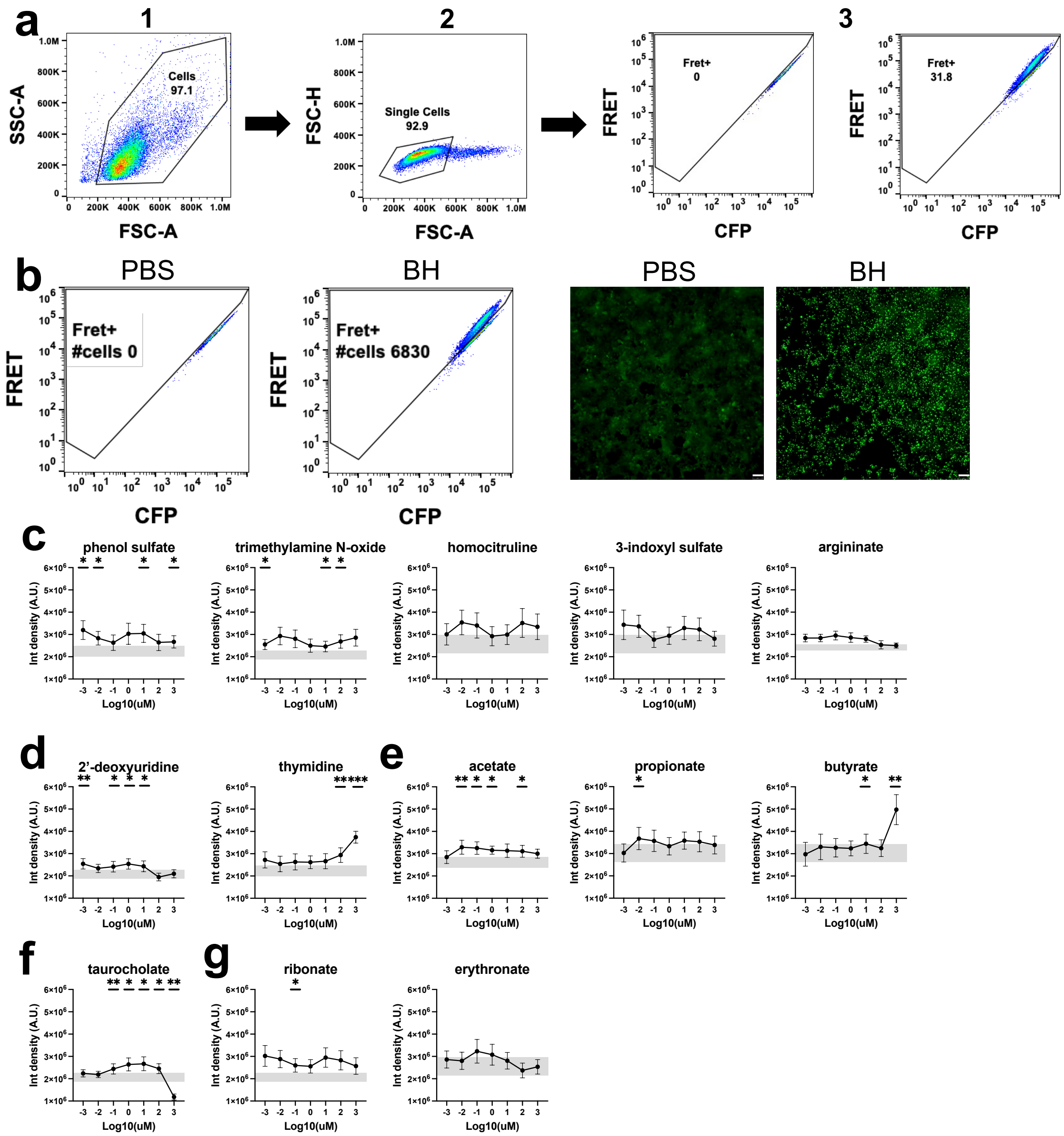

Fig. S8 | Validation of controls and additional metabolites for tau aggregation assay

(a) Flow-cytometry gating strategy. Gating hierarchy of a representative sample (positive control). From left to right : 1. in SSC-A vs FSC-A, identification of cell-like events; 2. in FSC-H vs FSC-A, identification of singlets (single cell signals); 3. in FRET vs CPF, identification of FRET positive cells (cells with aggregates). (b) Validation of seeding effect of brain homogenate (BH) from hTau mice on tau RD biosensor cells. (c-g) In vitro dose curves for metabolites that significantly increased tau aggregations compared to brain homogenate+PBS control. Shaded area represents average baseline (brain homogenate+PBS control) integrated density ± SEM (one-way ANOVA with Dunnett's test; n=6). Exact p values are provided in the Source Data. (c) amino acid metabolites (d) nucleotide metabolites (e) short chain fatty acids (f) lipid metabolites (g) carbohydrate metabolites. Results are expressed as mean ± SEM. *$p<0.05$, **$p<0.01$, ***$p<0.001$, ns=not statistically significant.

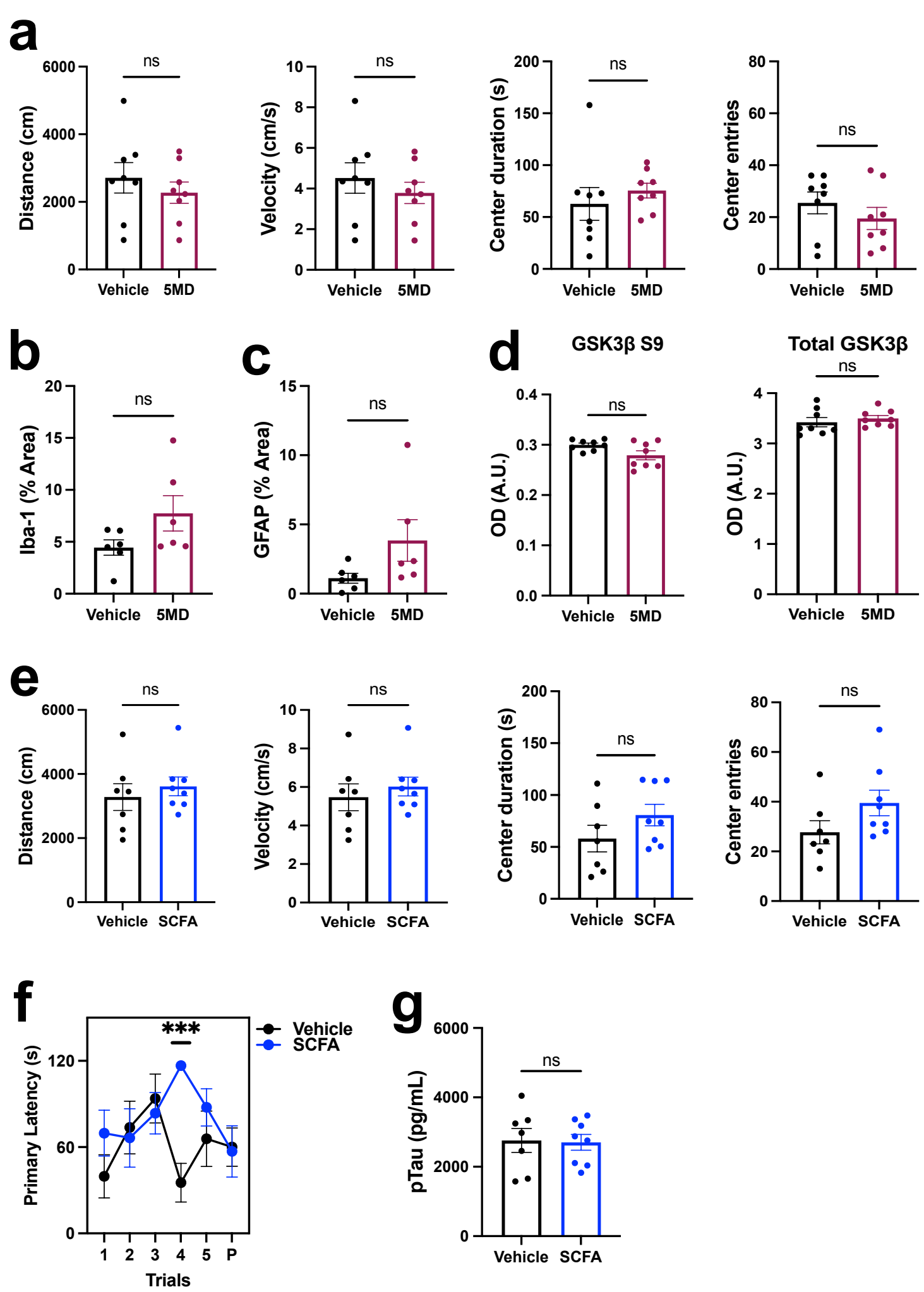

Fig. S9 | Behavioral, neuropathological, and biochemical alterations following microbial metabolite supplementation

(a) Open field test results for vehicle and 5MD treated mice. left to right: distance traveled, velocity, time spent in the center, number of entries to the center (two-tailed Welch's t test; vehicle n=8, 5MD n=8). (b) Quantification of Iba-1 in the hindbrain of 6-month-old vehicle and 5MD hTau mice (two-tailed Welch's t test; vehicle n=6, 5MD n=6). (c) Quantification of GFAP in the hindbrain of 6-month-old vehicle and 5MD hTau mice (two-tailed Welch's t test; vehicle n=6, 5MD n=6). (d) Relative levels of GSK3β and total GSK3β in 6-month-old brains of vehicle and 5MD hTau mice (two-tailed Welch's t test; vehicle n=8, 5MD n=8). (e) Open field test results for vehicle and short chain fatty acid (SCFA) treated mice (35.04 mM acetate, 3.154 mM propionate and 3.154 mM butyrate) left to right: distance traveled, velocity, time spent in the center, number of entries to the center (two-tailed Welch's t test; vehicle n=7, SCFA n=8). (f) Barnes maze performance for vehicle and mice treated with SCFA. Primary latency to enter target hole (two-way ANOVA with Tukey's test; p=0.0007; vehicle n=7, SCFA n=8). (g) Quantification of insoluble aggregated tau in 6-month-old brains of mice treated with SCFA (two-tailed Welch's t test; vehicle n=7, SCFA n=8). Results are expressed as mean ± SEM. ***p<0.001, ns=not statistically significant.
