# Supplementary material for: Select microbial metabolites promote tau aggregation in a murine tauopathy model
Source: Nat Commun. 2026 Jun 30;17:8161. doi: 10.1038/s41467-026-74775-6 (PMC13458332; doi:10.1038/s41467-026-74775-6)
Supplement: Supplementary file 4 [file 41467_2026_74775_MOESM2_ESM.pdf]

Description of Additional Supplementary Files

File Name: Supplementary Data 1
Description: 16S rRNA gene sequencing OTU table of WT and hTau.P301S mouse lines
Legend: Table displays 16S rRNA V4 gene sequencing OTU count table using DADA2 denoising and taxonomy assignment to Greengenes2 (v2024) database for WT and hTau mouse fecal samples.

File Name: Supplementary Data 2
Description: Metagenomics KEGG Ortholog (KO) RPKM count table of WT and hTau.P301S mice
Legend: Table information displays RPKM counts of KEGG Ortholog genes found in WT and hTau mouse fecal samples analyzed with HUMAnN2 pipeline.

File Name: Supplementary Data 3
Description: Metagenomics MetaCyc pathways relative abundance of WT and hTau.P301S mice
Legend: Table information displays relative abundance counts of MetaCyc metagenomic pathways found in WT and hTau mouse fecal samples analyzed with HUMAnN2 pipeline.

File Name: Supplementary Data 4
Description: Single nucleotide variants change in frequency in parallel across hTau.P301S mice
Legend: 6 genes with one or more single nucleotide variants (SNVs) undergoing frequency changes of $f \leq 0.2$ to $f \geq 0.8$ between timepoints across multiple hTau mice were detected in 4 bacterial species. For each gene, the number of SNVs undergoing extreme frequency changes, gene descriptions, KEGG IDs, and E.C. number are given, when available.

File Name: Supplementary Data 5
Description: Single nucleotide variants change in frequency in parallel across WT mice
Legend: 14 genes with one or more single nucleotide variants (SNVs) undergoing frequency changes of $f \leq 0.2$ to $f \geq 0.8$ between timepoints across multiple WT mice were detected in 6 bacterial species. For each gene, the number of SNVs undergoing extreme frequency changes, gene descriptions, KEGG IDs, and E.C. number are given, when available.

File Name: Supplementary Data 6
Description: Untargeted metabolomics for serum of WT and hTau.P301S mice
Legend: Red and green shaded cells indicate $p \leq 0.05$ (red indicates that the mean values are significantly higher for that comparison; green values significantly lower). Light red and light green shaded cells indicate $0.05 < p < 0.10$ (light red indicates that the mean values trend higher for that comparison; light green values trend lower).

File Name: Supplementary Data 7
Description: Untargeted metabolomics for brain of WT and hTau.P301S mice
Legend: Red and green shaded cells indicate $p \leq 0.05$ (red indicates that the mean values are significantly higher for that comparison; green values significantly lower). Light red and light green shaded cells indicate $0.05 < p < 0.10$ (light red indicates that the mean values trend higher for that comparison; light green values trend lower).

File Name: Supplementary Data 8
Description: Commonalities in metabolite trends between sera and brain for ABX treated and SPF hTau.P301S mice
Legend: List of significant metabolites that were found in both brain and serum. Up or down indicates whether the metabolite was found increased or decreased in ABX treated hTau mice compared to SPF hTau mice.

File Name: Supplementary Data 9
Description: MEGNA module data for metabolite network connections
Legend: Module-trait associations for age, gender, genotype, AD condition (AD vs. control), and microbiome (ABX vs. SPF) in brain or serum, respectively. Brain module MEc.1.16 (significantly associated with age and microbiome) and serum module MEc1.40 (significantly associated with microbiome) are highlighted as both modules contain the five cocktail metabolites (trimethylamine-N-oxide, phenol sulfate, 2'-deoxyuridine, thymidine, and 3-indoxyl sulfate), emphasizing the convergence of these two modules from different tissues at these five key metabolites. Statistical significance was determined by t-test or ANOVA. P-values were adjusted by Benjamini-Hochberg.

File Name: Supplementary Data 10
Description: Potential microbial pathways, enzymes, and microbes mediating 5MD in hTau.P301S mice
Legend: Microbial pathways and enzymes for 5MD metabolite production were manually curated using MetaCyc Metabolic Network Explorer. Microbe list was derived from manually cross-referencing potential pathways and enzymes with species-stratified HUMAnN results and filtering only pathways and enzymes present in hTau mice.
